# QTL mapping and genome-wide association study reveal two novel loci associated with green flesh color in cucumber

**DOI:** 10.1186/s12870-019-1835-6

**Published:** 2019-06-07

**Authors:** Kailiang Bo, Shuang Wei, Weiping Wang, Han Miao, Shaoyun Dong, Shengping Zhang, Xingfang Gu

**Affiliations:** Institute of Vegetables and Flowers, Chinese Academy of Agricultural Sciences, Beijing, 100081 China

**Keywords:** Cucumber, Green flesh, Chromosome rearrangements, QTL, GWAS

## Abstract

**Background:**

Green flesh color, resulting from the accumulation of chlorophyll, is one of the most important commercial traits for the fruits. The genetic network regulating green flesh formation has been studied in tomato, melon and watermelon. However, little is known about the inheritance and molecular basis of green flesh in cucumber. This study sought to determine the main genomic regions associated with green flesh. Three F_2_ and two BC_1_ populations derived from the 9110Gt (cultivated cucumber, green flesh color) and PI183967 (wild cucumber, white flesh color) were used for the green flesh genetic analysis. Two F_2_ populations of them were further employed to do the map construction and quantitative trait loci (QTL) study. Also, a core cucumber germplasms population was used to do the GWAS analysis.

**Results:**

We identified three indexes, flesh color (FC), flesh extract color (FEC) and flesh chlorophyll content (FCC) in three environments. Genetic analysis indicated that green flesh color in 9110Gt is controlled by a major-effect QTL. We developed two genetic maps with 192 and 174 microsatellite markers respectively. Two novel inversions in Chr1 were identified between cultivated and wild cucumbers. The major-effect QTL, *qgf5.1*, was identified using FC, FEC and FCC index in all different environments used. In addition, the same *qgf5.1*, together with *qgf3.1*, was identified via GWAS. Further investigation of two candidate regions using pairwise LD correlations, combined with genetic diversity of *qgf5.1* in natural populations, it was found that *Csa5G021320* is the candidate gene of *qgf5.1*. Geographical distribution revealed that green flesh color formation could be due to the high latitude, which has longer day time to produce the photosynthesis and chlorophyll synthesis during cucumber domestication and evolution.

**Conclusions:**

We first reported the cucumber green flesh color is a quantitative trait. We detected two novel loci *qgf5.1* and *qgf3.1*, which regulate the green flesh formation in cucumber. The QTL mapping and GWAS approaches identified several candidate genes for further validation using functional genomics or forward genetics approaches. Findings from the present study provide a new insight into the genetic control of green flesh in cucumber.

**Electronic supplementary material:**

The online version of this article (10.1186/s12870-019-1835-6) contains supplementary material, which is available to authorized users.

## Background

Fruit flesh color, an important feature for consumers’ choice, is a key trait of breeding [1]. Chlorophyll and carotenoid are the main pigments contribute to the flesh color formation [2]. Different composition and concentration of chlorophyll and carotenoid contribute to the red, orange, yellow, green, light green and white flesh color in Cucurbit fruits [3–5]. Chlorophyll and its natural or commercial derivatives have demonstrated to have antioxidant, and antimutagenic activity, and have function in modulating xenobiotic metabolizing enzymes, and induction of apoptotic events in cancer cell lines in vitro and in vivo experiments [6]. In addition, they have the ability to induce mammalian phase 2 proteins which protect cells against deleterious effect of oxidants and electrophiles [7]. Protective effects of chlorophyll and their watersoluble salts (chlorophyllin) against consequence of carcinogen exposure like aflatoxin were also confirmed in animals. Carotenoids including β–carotene, α-carotene, lutein, etc. are the important precursors of vitamin A, which is necessary for human health especially the eye health [8, 9].

Cucurbit fruits have rich variations in flesh color, especially in melon and water melon. In melon (*Cucumis melo* L.), the flesh color exhibited white, yellow and orange because of the accumulation of chlorophyll and carotenoid [4]. In the previous study, several genes/QTLs for flesh color in melon were reported. Hughes [10] and Imam et al. [11] first identified the green flesh (*gf*) gene and white flesh (*wf*) gene, respectively. Moreover, Clayberg [12] indicated that green and white flesh are recessive to orange, and also developed a genetic model for the inheritance among white, green and orange flesh. However, several follow-up studies didn’t confirm the above genetic model [13–15]. To understand the inheritance and gene control of flesh color more accurately, especially for the orange flesh color, the beta-carotene content combined with the flesh color were used for the analysis. Cuevas et al. [16] detected eight QTLs associated with the beta-carotene content used a set of 81 recombinant inbred lines (RIL) derived from ‘USDA 846–1’ and ‘Top Mark’. To detect more stable QTLs, Cuevas et al. [17] constructed a novel genetic map used a set of 116 F_3_ families derived from the ‘Q 3–2-2’ and ‘Top Mark’, and detected three QTLs related with beta-carotene content. Comparative analysis showed that three QTLs (*β-carE.6.1*, *β-carM.8.1* and *β-carM.9.1*) could be detected repeatedly in two different populations, which revealed these three QTLs more critical for the orange flesh formation in melon. In addition, Tzuri et al. [9] also identified a gene, *Cmor*, which was found to co-segregate with flesh color in melon. However, the molecular base and genetic control of flesh color formation in melon is still not clear.

In watermelon (*Citrullus lanatus*), different composition and concentration of carotenoids contribute to the red, orange, canary yellow, salon yellow and white flesh color [3]. Tadmor et al. [18] and Bang et al. [19] analysed the pigment components of different color flesh, and indicated that red flesh color results from lycopene, orange from prolycopene and rarely from β-carotene, canary yellow from xanthophylls and β-carotene, salmon yellow from pro-lycopene, which suggested the flesh color of watermelon is a complex trait. Briefly, white flesh is epistatic to canary yellow, canary yellow is epistatic to coral red, canary yellow is dominant to red and orange [3, 20]. For the gene/QTL study, Hashizume et al. [21] detected two flesh color QTLs in a biparental F_2_ population derived from H-7 (red flesh) and SA-1 (white flesh). Liu et al. [22, 23] identified only one QTL on chromosome 4 used a F_2_ population derived from LSW-177 (red flesh) and COS (yellow flesh). Branham et al. [24] combined visual color phenotyping with genotyping-by-sequencing of an F_2:3_ population derived from NY0016 (orange flesh) and EMB (canary yellow flesh) and detected a major locus on Chr1, which was associated with β-carotene content. Recently, Zhang et al. [25] reported that chromoplast development plays a crucial role in controlling carotenoid content in watermelon flesh, and detected a key gene *ClPHT4;2* which was up-regulated during flesh color formation in watermelon. Thus, it is likely that several different genes and biochemical pathways affect the pigment accumulation during watermelon flesh color formation.

In cucumber, Qi [26] first described the semi-wild Xishuangbanna cucumber (*Cucumis sativus* L. *var. xishuangbannanesis* Qi et Yuan) has orange flesh color, which is due to the accumulation of high level of β-carotene in mature fruits [27]. Inheritance analysis indicated that two recessive genes control orange mesocarp, while a single recessive gene controlled orange endocarp [28]. Then, Bo et al. [27] first mapped the *ore* (orange endocarp) gene on Chr3. Another colored cucumber is PI200815 originate from Myanmar, which has the yellow flesh color in the mature fruits [29]. Kooistra [30] analyzed the inheritance of yellow fruit flesh in cucumber and suggested that flesh color (including orange, yellow, dingy white, and intense white) was determined by two genes. Lu et al. [29] reported that yellow flesh was controlled by a recessive gene (*yf*) and finished the initial mapping. However, the above orange and yellow flesh color only appeared in the mature stage, which is difficult to be used for cucumber production. In the modern cucumber breeding, green flesh is already one of the most important quality traits. While there are very few reports regarding the green flesh in cucumber.

In the present study, three indexes (flesh color, flesh extract color and flesh chlorophyll content) were used to identify the green flesh. Three F_2_, one BC_1_P_1_ and one BC_1_P_2_ populations were used to analyze the inheritance of flesh color, and the green flesh locus was mapped by using two F_2_ populations in two environments. In addition, we detected two locus *qgf3.1* and *qgf5.1* using GWAS. The *qgf5.1* is consistent with the major QTL detected in two F_2_ populations. To further investigate the domestication history of green flesh, we did the world map distribution analysis with 115 core cucumber germplasms (CG), which indicated that the higher latitude region has more green flesh cucumbers. This study thus provides important insights into the green flesh color formation in cucumber.

## Results

### Phenotypic variation of FC, FEC and FCC in F_2_ and BC_1_ populations

**Fig. 1:**
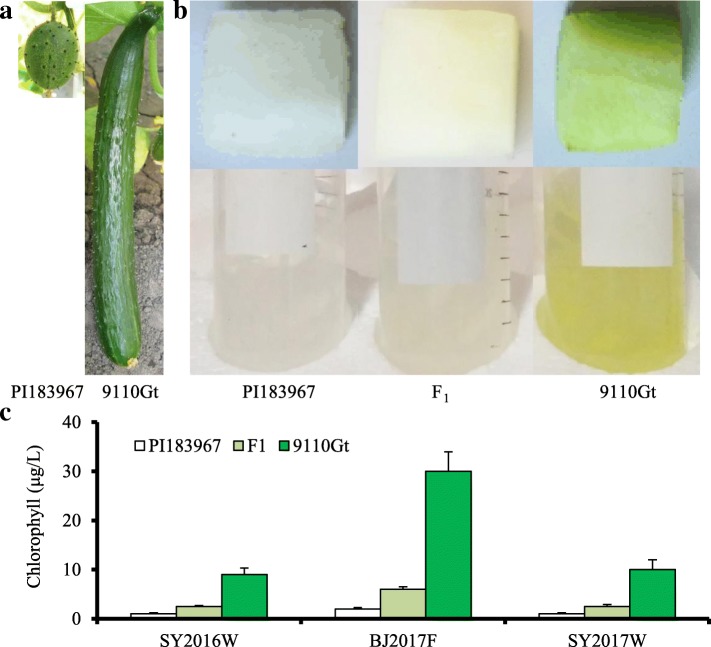
Commercial fruit, flesh color and chlorophyll content among PI183967, 9110Gt and their F_1_ in three experiments. **a** Commercial fruits of two parent lines. **b** Flesh color and flesh extract color among two parents and their F_1_. PI183967 exhibit wild flesh, 9110Gt exhibit green flesh. **c** The flesh chlorophyll content among two parents and their F1 in SY2016W, BJ2017F and SY2017W. *SY2016W* Sanya 2016 winter, *BJ2017F* Beijing 2017 fall, *SY2017W* Sanya 2017 winter

**Fig. 2:**
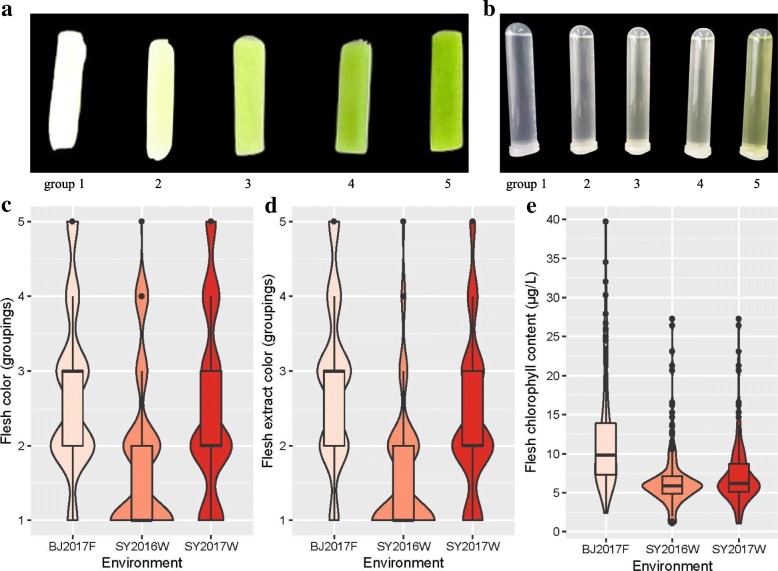
Violin and box plots depicting phenotypic distribution of flesh color, flesh extract color and flesh chlorophyll content among SY2016W, BJ2017F and SY2017W experiments. **a** Flesh color of the five groups in the progeny population. **b** Flesh extract color of the five groups in the progeny population. **c** Violin and box plots depicting flesh color. **d** Violin and box plots depicting flesh extract color. **e** Violin and box plots depicting flesh chlorophyll content

Phenotypic data of FC, FEC and FCC were collected from the two parents, their F_1_, three F_2_, one BC_1_P_1_ and one BC_1_P_2_ populations in five experiments over two years (Fig. 1, Additional file 1: Table S1). The FC and FEC were categorized into five color groupings in above populations (Additional file 1: Figures S1 and S2) using the grade scale (Fig. 2a-b). The frequency distributions of FC, FEC and FCC among the populations from different experiments are illustrated in Fig. 2c-e and Additional file 1: Figure S3. All observed distributions of FC and FEC in three F_2_ populations showed a clear bimodal distribution, which suggested that the flesh color was controlled by a major QTL. The average of FC, FEC and FCC in BJ2017F experiment was found to be higher than that of the SY2016W and SY2017W experiments (Table 1, Fig. 2c-e). The reason could be that longer day time in Beijing promotes chlorophyll synthesis during the fruit development than Sanya (See discussion).

**Table 1:** Phenotypic means and range of FC^a^, FEC and FCC of PI183967, 9110Gt, their F_1_ and F_2_ from 3 experiments (SY2016W^b^, BJ2017F and SY2017W)

Traits	PI183967	9110Gt	F1	SY2016W		BJ2017F		SY2017W	
	Mean ± SD	Mean ± SD	Mean ± SD	Mean ± SD	Range	Mean ± SD	Range	Mean ± SD	Range
FC (grade)	0 ± 0	4 ± 0	1 ± 0	1.80 ± 1.00	1–5	2.72 ± 1.10	1–5	2.51 ± 1.23	1–5
FEC (grade)	0 ± 0	4 ± 0	1 ± 0	1.58 ± 0.84	1–5	2.82 ± 1.08	1–5	2.36 ± 1.06	1–5
FCC (μg/L)	1.33 ± 0.12	16.33 ± 2.61	3.67 ± 0.47	6.55 ± 3.63	1.13–27.57	11.85 ± 6.97	2.40–39.70	7.42 ± 4.34	1.08–27.27

^a^*FC* Flesh color, *FEC* Flesh extract color, *FCC* Flesh chlorophyll content

^b^*SY2016W* Sanya 2016 winter, *BJ2017F* Beijing 2017 fall, *SY2017W* Sanya 2017 winter

The FC, FEC and FCC correlations among multiple environments were examined for each population and the Spearman’s rank correlation coefficients (*r*_*s*_) are presented in Table 2. Strong positive correlations were observed among FC, FEC and FCC in different environments in each population, which suggested the green flesh phenotype is caused by the higher chlorophyll content in cucumber fruit. Interestingly, for the SY2016W and SY2017W experiments, the average of FC, FEC and FCC in SY2016W is lower than that in SY2017W, which probably because of the small amount of chlorophyll degradation during long-distance transportation from Sanya to Beijing (about 7 days). In SY2016W experiment, all the phenotypic data were collected in Beijing. While in SY2017W experiment, we collected all the data in Sanya using the fresh cucumber flesh. Despite this, the QTL detected with the SY2016W data was very consistent with that identified with other data sets (see below).

**Table 2:** Spearman’s rank correlation coefficients among different environments in the 9110Gt × PI183967 F_2_ population

	SY2016W^a^		BJ2017F		SY2017W	
	FEC	FCC	FEC	FCC	FEC	FCC
FC^b^	0.669^**^	0.572^**^	0.838^**^	0.650^**^	0.881^**^	0.780^**^
FEC		0.668^**^		0.776^**^		0.861^**^

^a^*SY2016W* Sanya 2016 winter, *BJ2017F* Beijing 2017 fall, *SY2017W* Sanya 2017 winter

^b^*FC* Flesh color, *FEC* Flesh extract color, *FCC* Flesh chlorophyll content

**P < 0.01

To summarize, despite the different environments and scoring scales in the five phenotyping experiments, data collected from these trials were highly correlated, consistent, and of good quality, which provided a solid foundation for subsequent QTL analysis.

### Linkage map construction

We screened the cucumber SSR primer pairs between PI183967 and 9110Gt and identified 201 polymorphic ones for genetic map construction. The resulting genetic map is illustrated in Additional file 1: Figures S4 and S5, and the main statistics of the map are presented in Table S2. Detailed information (marker names, map location, 9930 draft genome assembly locations and primer sequences) of two maps was provided in Additional file 1: Table S3 and S4.

We developed two high-density maps using two F_2_ populations, respectively. The 234 F_2_ map included 192 markers that spanned 922.3 cM with an average marker interval of 4.95 cM. The 125 F_2_ map comprised 174 markers that spanned 901.1 cM with an average marker interval of 5.67 cM.

According to the 9930 genome (V2.0) anchored by these markers, these two maps seemed to physically cover the majority of the cucumber genome. Therefore, these two genetic maps were suitable for the QTL mapping analysis.

### Chromosome rearrangements between cultivated and wild cucumbers

**Fig. 3:**
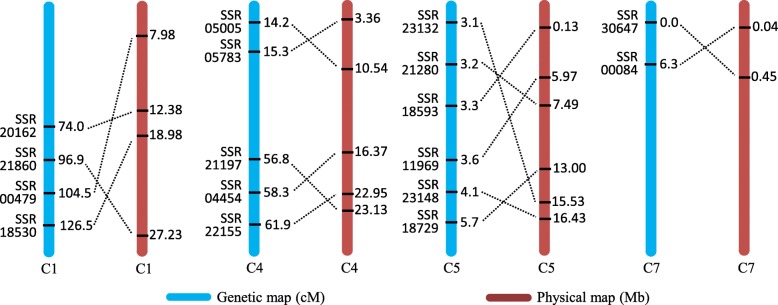
Putative structural rearrangements in chromosomes 1, 4, 5 and 7 between wild (*C. sativus* var. *hardwickii*) and cultivated (*C. sativus* var. *sativus*) cucumbers. Structural changes were inferred based on the markers order in genetic map (in cM, left, blue color) and their physical location in 9930 draft genome V2 (in Mb, right, red color). Only representative SSR markers and their location involved in the putative rearrangements are listed. *Dotted lines* connect the same markers between two maps under comparison

To investigate possible chromosome structural rearrangements between the cultivated (*C. s.* var. *sativus*) and wild (*C. s.* var. *hardwickii*) cucumbers, we aligned the two wild cucumber originated maps developed herein with the 9930 genome (V2.0). Several typically shared markers in two maps presented in Additional file 1: Figures S3 and S4 were used for the chromosome by chromosome alignment. Putative structural rearrangements represented by SSR markers between cultivated cucumber and wild cucumber are illustrated in Fig. 3.

In Chr1, 4, 5 and 7, there were two, two, three and one blocks, respectively, in which the orders of molecular markers were inconsistent with the physical location suggesting possible inversions between the cultivated and wild cucumbers. It is known that two, three, and one inversion differentiated the Chr4, 5, and 7 of cultivated and wild cucumbers [31]. The locations of the rearrangements (on Chr4, 5 and 7) identified from the present study were largely consistent with those found between the cultivated and wild cucumbers. Interestingly, we found a novel putative inversion in Chr1 (Fig. 3). The above putative inversions in wild cucumber could be the reason to promote cucumber evolution in order to adapt different environments (See below).

### QTL mapping of FC, FEC and FCC

**Fig. 4:**
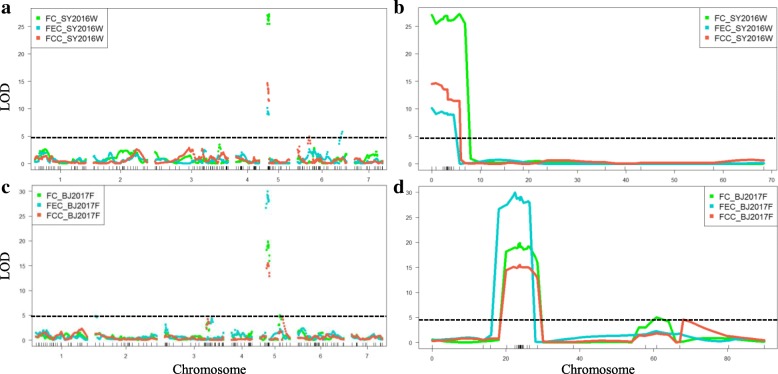
Whole genome view of QTL locations for flesh color, flesh extract color and flesh chlorophyll content in 234 F_2_ (**a**, **b**) and 125 F_2_ (**c**, **d**) populations detected in two experiments (SY2016W and BJ2017F) based on CIM model in R/qtl. For each population, the X axis represents linkage map of seven chromosomes, and the Y axis is LOD scores; the horizontal line represents LOD threshold obtained with 1000 permutation tests (*P* = 0.05)

We conducted QTL analysis using the CIM approach with FC, FEC and FCC phenotypic data for each experiment. Initial whole genome QTL mapping was conducted with a window size of 25 cM because of a few large gaps (> 10 cM) in the genetic maps (Additional file 1: Figures S4, S5). The LOD threshold to declare significance of QTL for each trait was determined with 1000 permutation tests (*P* = 0.05). All green flesh related QTLs detected in the present study were illustrated in Fig. 4a, c. The QTL information for FC, FEC and FCC are shown in Table 3. Figure 4b, d showed the major QTL detected in two populations. We total detected six QTLs by using FC, FEC and FCC three indexes.

**Table 3:** QTL for flesh color (*fc*), flesh extract color (*fec*) and flesh chlorophyll content (*fcc*) detected with two F_2_ populations (9110Gt × PI183967) in two experiments (SY2016W: Sanya 2016 winter, BJ2017F: Beijing 2017 fall)

Populations	Environments	Consensus	Supporting	Chr	QTL peak	Peak LOD	1.5 LOD interval		Phenotypic
		QTL	QTL		(cM)	score	left (cM)	right (cM)	variation (%)
234 F_2_	SY2016W	*qgf5.1*	*fc_5.1*	5	SSR18729 (5.7)	27.2	SSR13340 (0.0)	SSR14611 (6.9)	38.3
			*fec_5.1*	5	SSR13340 (0.0)	10.1	SSR13340 (0.0)	SSR18729 (5.7)	22.2
			*fcc_5.1*	5	SSR07284 (0.9)	14.6	SSR13340 (0.0)	SSR14269 (3.3)	23.9
125 F_2_	BJ2017F	*qgf5.2*	*fc_5.2*	5	SSR16032 (23.7)	19.3	SSR14611 (20.1)	SSR07284 (28.5)	57.6
			*fec_5.2*	5	SSR16691 (22.5)	29.5	SSR14611 (20.1)	SSR07284 (28.5)	63.9
			*fcc_5.2*	5	c5.loc22 (22.0)	21.0	SSR17464 (15.8)	SSR07284 (28.5)	49.1

We examined the relationships of QTL detected with three different phenotypic indexes (FC, FEC and FCC) in SY2016W and BJ2017F experiments. The results are presented in Table 3 and Fig. 4. All three datasets detected QTL in Chr5. The 1.5-LOD intervals and peak locations of QTL for FC, FEC and FCC were overlapped, suggesting that they probably belonged to the same QTL although the LOD support value and the effects were somewhat different (Table 3). In SY2016W experiment, the QTL with largest effect, *qgf5.1* (*R*^*2*^ = 22–38%), was identified using FC, FEC and FCC three different indexes, with a highly consistent peak on the genetic map at 5.7 cM (Fig. 4b and Table 3). In BJ2017F experiment, the QTL with largest effect, *qgf5.2* (*R*^*2*^ = 49–64%), was also identified using FC, FEC and FCC three different indexes, with a highly consistent peak on the genetic map at 22.5 cM (Fig. 4d and Table 3).

In brief, two major-effect QTLs *qgf5.1* and *qgf5.2* were detected by using three indexes in two experiments, which indicated that *qgf5.1* and *qgf5.2* two loci mainly control the green flesh formation in cucumber. While the *qgf5.1* and *qgf5.2* QTLs are located on the different locations on Chr5, we still believe they are the same QTL because these two QTLs located in the inversion region on Chr5 (Fig. 3). That also suggested the green flesh could be a domestication trait during cucumber evolution.

### Evolution and geographical distribution analysis of green flesh in a natural population

**Fig. 5:**
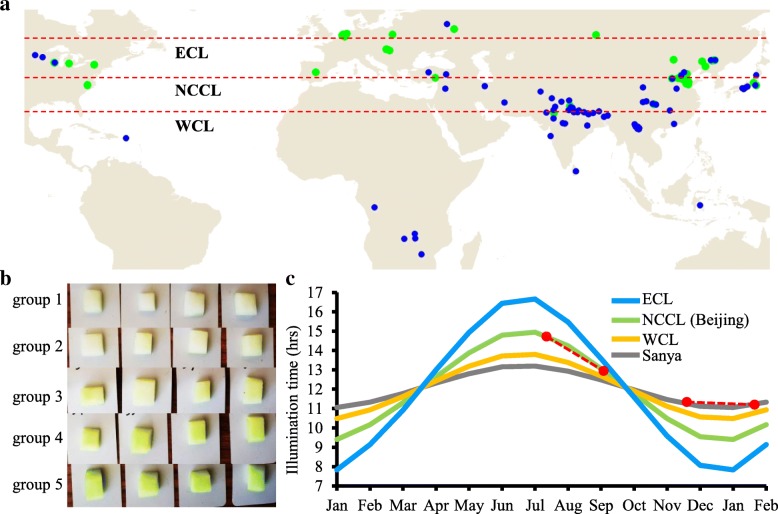
Green flesh worldwide geographic distribution and illumination time of the typical location. **a** Distribution of 115 cucumber CG lines in different continents based on flesh color data. To understand the distribution easier, we defined group 1, 2 and 3 as white flesh, group 4 and 5 as green flesh. **b** Part CG lines flesh color showing five different groups. **c** Illumination time of the wild cucumber latitude (WCL), European cucumber latitude (ECL), North China cucumber latitude (NCCL, same with the experiment location in Beijing) and the experiment location in Sanya. The left red dashed line means the illumination time pattern during the cucumber growing season in Beijing. The right dashed line means the illumination time pattern during the cucumber growing season in Sanya

The flesh color of all 115 cucumber CG lines was categorized into five groups based on flesh color (Fig. 5b). To understand the flesh color distribution in the world easier, we defined group 1, 2 and 3 as white flesh (chlorophyll content < 11 μg/L), group 4 and 5 as green flesh (chlorophyll content > 11 μg/L). The approximate geographical coordinates (altitude and latitude) of the origin of each line were used to plot the 115 lines on a world map (Fig. 5a). We found almost all the European cucumber has the green flesh. For the North China cucumber, around half presented green flesh. However, in the South China and India type cucumber, few accessions with the green flesh. To further ascertain the domestication path of green flesh in cucumber, we defined three latitudes on the distribution map: wild cucumber latitude (WCL), North China cucumber latitude (NCCL), and European cucumber latitude (ECL) (Fig. 5a). Interestingly, the higher latitude, the more cucumbers presented green flesh, which suggesting the green flesh could be affected by the environment during domestication and evolution. The reasonable explanation should be the illumination time in higher latitude area is longer than that in lower latitude. Four critical illumination times over one year were showed in Fig. 5c. We found the illumination time at ECL is significantly longer than that in WCL from May to October. Longer illumination time will promote the chlorophyll synthesis in cucumber fruit, which could be the reason of green flesh formation. We also observed that SCCL has higher genetic diversity for cucumber fruit flesh color, which may attribute to its extensive history of domestication or diversity selection in cucumber breeding.

### Candidate gene identification of the green flesh using GWAS

**Fig. 6:**
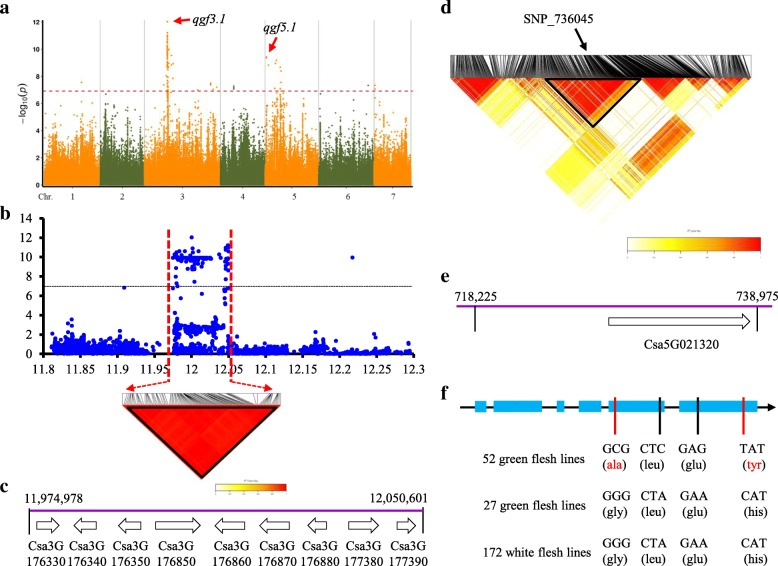
GWAS for flesh chlorophyll content and identification of the causal gene for the peak on chromosome 3 and 5. **a** Manhattan plot for flesh chlorophyll content. Dashed line represents the significance threshold (−log_10_ *P* = 7.00). Arrowheads indicate the position of strong peaks. **b** Local Manhattan plot (top) and LD heatmap (bottom) surrounding the peak on chromosome 3. Dashed lines indicate the candidate region (~ 75 kb) for the peak. **c** Nine genes were predicted in the *qgf3.1* candidate region. **d** LD heatmap based on the SNPs located on the 100 Kb region surrounding the SNP_736045 peak. **e** The candidate gene of *qgf5.1* was detected in the block region (~ 20 kb). **f** SNP variation in the natural population. 251 cucumber lines were used for the candidate gene alignment. The red color means the change of SNP results the amino acid change

We detected a major QTL in Chr5 using two F_2_ populations, while it is difficult to do the fine mapping work because the QTL is located on the inversion region. To verify the locus on Chr5 and detect more novel locus related to the green flesh, 115 CG lines were used to do the GWAS analysis. Using the general linear model, two locus, *qgf3.1* and *qgf5.1*, were detected that consistently exceeded a significant threshold (−log_10_*P* ≥ 7.0) (Fig. 6a). For the *qgf3.1*, a candidate region was estimated to extend from 11.975 Mb to 12.051 Mb (~ 76 kb) on Chr3 using pairwise LD correlations (*r*^2^ ≥ 0.6) (Fig. 6b). According to the Cucumber Genome Browser (http://cucurbitgenomics.org/organism/2), nine annotated genes are located in the *qgf3.1* candidate region (Fig. 6c). For the *qgf5.1*, we observed that the SNP_736045 showed the strongest association with green flesh (Fig. 6d). We extended 100 Kb near the SNP_736045 for pairwise LD correlation analysis. A small candidate region was estimated to extend from 718.225 Kb to 738,975 Kb (~ 20 kb) on Chr5, which included one annotated gene (Fig. 6e). To verify the candidate gene of *qgf5.1*, we did an alignment among 251 natural cucumber lines. The results showed that four unique SNPs that were associated with green flesh (Fig. 6f). Among the four SNPs, two were located on the fifth exon and the other two were located on the sixth exon. The first and fourth SNP resulted in an amino acid substitution from G (gly) to A (ala) and H (his) to Y (tyr), respectively (Fig. 6f). Interestingly, the SNP variation of green flesh line is not consistent with the phenotype completely, which indicated that the green flesh in natural cucumber could be controlled by multiple genes (such as the loci *qgf3.1*).

## Discussion

### Inheritance and QTL/gene of the cucumber flesh color

In the present work, two parents (P1: PI183967; P2: 9110Gt), their F_1_, three F_2_, one BC_1_P_1_ and one BC_1_P_2_ populations in five experiments over two years were used to study the inheritance of FC, FEC and FCC in cucumber. The FC, FEC and FCC phenotypic data had a significant positive correlation for the same population in different experiments (Table 2). The frequency distributions of FC, FEC and FCC among the three F_2_ populations were bimodal rather than normal (Fig. 2), especially for the FC and FEC, suggesting that green flesh color is controlled by a major QTL. A number of other cucumber flesh color trait have been also identified, such as orange endocarp, orange mesocarp and yellow flesh. Cuevas et al. [28] reported the single gene model for the orange endocarp using F_2_ populations. Bo et al. [27] also confirmed this result using 124 recombinant inbred lines in three different environments.

Regarding the inheritance of orange mesocarp in cucumber, Navazio [32] presented single gene model, while Cuevas et al. [28] showed two gene model. The difference probably results from the population sizes [Navazio [32] = 46 F_2_ progenies versus Cuevas et al. [28] = 111 F_2_ and 51 BC_1_P_2_ progenies] and/or the growing environment. For the yellow flesh, Lu et al. [29] showed that a single recessive gene controlled the yellow flesh using a large F_2_ population. The above genetic analysis indicated that cucumber flesh color is mostly a quality trait, which suggests that the specific genes mutant result in the flesh color formation during cucumber evolution and domestication.

### Chromosome differentiation of cultivated and wild cucumbers

Based on the taxonomic studies, cucumber can be divided into four different botanical variants: the cultivated cucumber, the wild cucumber [33, 34], the Sikkim cucumber [35], and the Xishuangbanna cucumber [26, 36]. Among of them, the wild cucumber has a large difference with other cucumbers on the morphological variation. Thus, the cucumber can be also divided into two extremities: the wild cucumber and the cultivated cucumber [37, 38].

Several previous studies reported the chromosome rearrangements between the wild cucumbers and cultivated cucumbers [31, 39, 40]. In order to get more information about the chromosome rearrangements between the wild and cultivated cucumber, we did a global analysis in this study. We detected two, two, three and one chromosome rearrangements in Chr1, 4, 5 and 7, respectively (Fig. 3). Yang et al. [31] identified six inversions in Chr4, 5, and 7 between wild and cultivated cucumber. Compared with the previous study, we found a novel putative inversion in Chr1. In plants, the inversions play an important role in the domestication and evolution [41–43]. Moreover, the previous studies also identified the same six inversions in the cultivated cucumber [31, 40, 44, 45], which suggested that the six inversions are common to wild and cultivated cucumbers. However, the two novel inversions detected in the present study between cultivated and wild cucumbers could be unique for the wild cucumber and further research is needed.

### Green flesh, chlorophyll metabolism and photosynthesis

In the present study, FC, FEC and FCC three indexes were used to define the green flesh color. QTL mapping results showed that all these three traits shared the same locus (Fig. 4), which suggesting that cucumber green flesh formation could because the chlorophyll accumulation during fruit development. Moreover, we found the cucumber with higher chlorophyll content often origin from the higher latitude area (Fig. 5a). The reasonable explanation is that the higher latitude area has longer illumination time (Fig. 5c), which can maintain longer photosynthesis and improve the chlorophyll synthesis. The chlorophyll content, as a critical feature of unripe fruits, affects the nutritional components and flavor of ripe fruits. Moreover, the link between chlorophyll content and photosynthesis in fruit tissues has been illuminated by a variety of studies [46, 47].

Tomato (*Solanum lycopersicum*) is a typical fruit with obvious chlorophyll metabolism at maturation and has been used as a model for chlorophyll metabolism studies. The regular tomato shows red flesh color at the ripening stage, while the *green-flesh* (*gf*) mutant of tomato still inhabited green flesh at the ripening stage because of the lack of chlorophyll degradation in *gf* mutant [48]. Moreover, Akhtar et al. [49] found that the leaves of the *gf* mutant also showed stay-green phenotype, which means the *GF* gene plays the same role both in leaves and fruits. In the *gf* mutant fruits, lots of chlorophyll still remain in the ripe fruit, suggesting that chlorophyll degradation is defective in the mutant material. And it might due to the amount of the chloroplast thylakoid grana existed in the plastids of ripe fruits [50]. Roca et al. [51] discussed the carotenoid biosynthesis in the *gf* mutant and showed that the carotenogenesis can be slowed in mutant lines. In addition, several genes were reported to affect the photosynthesis and chloroplast development for the chlorophyll metabolism in tomato. *LeHY5* is positive for the fruit pigment accumulation while *LeCOP1LIKE* gene is a negative regulator [52]. Tomato *high pigment-2*^*dg*^ mutant showed a highly significant increase in chloroplast size compared with the regular tomato [53]. Rohrmann et al. [54] identified AP2-EREBP, AUX/IAA, C2C2 etc. transcription factors regulated the chlorophyll level. Then, Waters et al. [55] confirmed that the *GLK* genes can influence chloroplast development. Recently, *APRR2-Like* genes were reported to increase plastid number, area, and enhance the chlorophyll levels in immature tomato fruits [56].

However, very few genes controlling fruits chlorophyll content were reported in cucumber. The candidate region/gene in the present study provides important clues for future fine mapping and cloning of these green flesh color loci. Nine and one candidate genes were predicted for the *qgf3.1* and *qgf5.1* loci by GWAS, respectively. Based on the predicted function, the candidate genes play roles in carbohydrate metabolic process (*Csa3G176330*), ribosome biogenesis (*Csa3G176340*), histone peptidyl-prolyl isomerization (*Csa3G176850*), glycolytic process (*Csa3G176870*), chloroplast envelope (*Csa3G177380*) and chloroplast stroma process (*Csa5G021320*), etc. Thus, the *Csa3G177380* and *Csa5G021320* are the most likely candidate genes for *qgf3.1* and *qgf5.1* loci. The SNPs developed in this work are also useful for marker-assisted selection in breeding for green flesh cucumber. While, due to the F_2_ population employed in the present study can’t be used to collect phenotypic data in multiple years/environments. To verify the green flesh loci target region and get more accurate and stable loci, we are developing the recombinant inbred lines (RILs). The next plan will verify the candidate genes using RILs and more natural accessions, and will also clone the genes for functional analysis.

## Conclusions

We reported the cucumber green flesh color is a quantitative trait. Two novel loci *qgf5.1* and *qgf3.1*, which regulate the green flesh formation in cucumber, were identified using QTL mapping and GWAS approaches. We also identified several candidate genes for further validation using functional genomics or forward genetics approaches. In addition, two novel chromosome rearrangements were detected in Chr1 between cultivated and wild cucumber.

## Methods

### Plant materials and mapping populations

Two inbred lines, 9110Gt (P_1_) and PI183967 (P_2_) were used as the parental lines to develop three F_2_ (234, 125 and 140 individuals, respectively), one BC_1_P_1_ (78 individuals) and one BC_1_P_2_ (77 individuals) populations for genetic analysis in the present study. 9110Gt was derived from the cross between a European greenhouse hybrid and a Northern Chinese line with dominant European glasshouse cucumber genetic background, which has green flesh color and high chlorophyll content (Fig. 1). PI183967 is a typical wild cucumber, which has white flesh and low chlorophyll content (Fig. 1). Among the above populations, 234 and 125 F_2_ individuals were used for genetic map construction and QTL mapping. For association analysis, 115 cucumber core germplasm (CG) lines were identified [57]. All the material seeds used in the present study were provided by the Cucumber Research Group of the Institute of Vegetable and Flowers Chinese Academy of Agricultural Science.

### Phenotypic data collection and analysis

For the genetic analysis study, phenotypic data of flesh color were collected in three environments over 2 years (2016, 2017) at two locations, which were designated as SY2016W, BJ2017F and SY2017W, respectively. SY2016W and SY2017W were conducted at the Sanya Research Station (109°60′ N, 18°29′ E), Hainan, China in 2016 and 2017 winter, respectively. BJ2017F was performed at the Nankou Research Station (116°10′ N, 40°22′ E), Beijing, China in 2017 fall. The two parental lines and their F_1_ were included in all screening tests.

For the GWAS study, phenotypic data of flesh color were collected in two environments, which were designated as CG2017S and CG2017F. CG2017S and CG2017F were conducted at the Nankou Research Station (116°10′ N, 40°22′ E), Beijing, China in 2017 spring and fall.

To identify the phenotype more accurately, we used three indexes: flesh color (FC), flesh extract color (FEC) and flesh chlorophyll content (FCC). For the FC identification, about one cubic centimeter flesh block was extracted from each fruit (Additional file 1: Figures S1 and S2). All the blocks were categorized into five color groupings (group 1 to group 5) based on the visual measurement. For the FEC identification, 2 g flesh sample of each fruit was put into 50-ml tubes with 40 ml extraction solution (95% alcohol) and kept in dark for 24 h. Then, the extract color was also categorized into five color groupings (group 1 to group 5) based on the visual measurement. The method of chlorophyll content measurement followed Tang et al. [58]. All three indexes were used for the QTL mapping study.

In each experiment, the FC/FEC/FCC was collected from three commercial mature cucumber fruits in the same individual. Statistical analysis of phenotypic data was performed using SAS v9.3 (SAS Institute Inc., Cary, NC, USA). Pearson’s correlation coefficients among different traits for each population were estimated with the PROC CORR function based on the grand mean of each experiment.

### Linkage map development

Cucumber SSR markers described in Ren et al. [39], Cavagnaro et al. [59], and Yang et al. [31] were used to screen for polymorphisms in crosses between 9110Gt and PI183967. Polymorphic markers were used to genotype the two F_2_ populations (234 and 125 individuals). All markers were tested against the expected segregation ratio of 1:2:1 or 3:1 using Chi-squared tests (*χ*^*2*^, *P* < 0.05). Linkage analysis was carried out with JoinMap 4.0. Genetic map was developed with the regression mapping method and Kosambi mapping function.

DNA extraction, PCR amplification of molecular markers, and gel electrophoreses were performed as described by Li et al. [60].

### QTL analysis

QTL analysis was performed with the R/qtl software package (http://www.rqtl.org/) with composite interval mapping (CIM) method [61, 62]. Genome-wide LOD threshold values (*P* < 0.05) for declaring the presence of QTLs were determined using 1000 permutations. The refined significant QTLs were assessed for the percentage of phenotypic variations (*R*^*2*^) explained. The support intervals for these QTLs were calculated using a 1.5-LOD drop interval. QTL naming conventions followed Bo et al. [36]. For example, *qgf5.1* designated the first QTL for green flesh on cucumber Chr5.

### Geographical distribution analysis and day length calculation

The geographical information of 115 CG lines was described in the previous study [57, 63]. DIVA-GIS software was used to construct the geographical map followed by Bo et al. [64]. The day length was calculated by subtracting sunrise time from sunset time. The sunset/sunrise time data among Beijing China, Sanya China, India and Netherland was downloaded from the website http://richuriluo.qhdi.com.

### Genome-wide association analyses of flesh chlorophyll content

A general linear model (GLM) was used for association tests, with an estimated relatedness matrix as covariate. A total of 3,877,848 SNPs were used for this analysis [65]. GWAS was conducted, and the genome-wide lowest *P* value was recorded. The 5% lowest tail was taken from the 200 recorded minimal *P* values as the threshold for genome-wide significance. The Manhattan map for GWAS was generated by using the R package qqman [66].

## Additional file

Additional file 1: **Figure S1.** Flesh color of five groups in the F_2_ population. **Figure S2.** Flesh color of five groups in the backcross population. a Flesh color distribution in BC_1_P_1_ population (P_1_ is the green flesh parent 9110Gt). b Flesh color distribution in BC_1_P_2_ population (P_2_ is the white flesh parent PI183967). **Figure S3.** Violin and box plots depicting phenotypic distribution of flesh color, flesh extract color and flesh chlorophyll content in SY2017W experiment. a Violin and box plots depicting flesh color. b Violin and box plots depicting flesh extract color. c Violin and box plots depicting flesh chlorophyll content. **Figure S4.** Linkage map constructed using a 234 F_2_ population in SY2016W experiment. **Figure S5.** Linkage map constructed using a 125 F_2_ population in BJ2017F experiment. **Table S1.** Summary of populations used for phenotypic data collection, QTL mapping and GWAS analysis. **Table S2.** Statistics of two linkage maps. **Table S3.** Information of markers mapped with 234 9110Gt×PI183967 F_2_ population. **Table S4.** Information of markers mapped with 125 9110Gt×PI183967 F_2_ population. **Table S5.** Information of the CG lines used in this study. (PDF 663 kb)

## Data Availability

All data generated or analysed during this study are included in this published article and its supplementary information files.

## Abbreviations

CG: Core germplasms
FC: Flesh color
FCC: Flesh chlorophyll content
FEC: Flesh extract color
QTL: Quantitative trait loci
SNP: Single nucleotide polymorphism
SSR: Simple sequence repeat
