# Supplementary material for: QTL mapping and genome-wide association study reveal two novel loci associated with green flesh color in cucumber
Source: BMC Plant Biol. 2019 Jun 7;19:243. doi: 10.1186/s12870-019-1835-6 (PMC6556036; doi:10.1186/s12870-019-1835-6)
Supplement: Supplementary file 1 — Figure S1. Flesh color of five groups in the F2 population. Figure S2. Flesh color of five groups in the backcross population. a Flesh color distribution in BC1P1 population (P1 is the green flesh parent 9110Gt). b Flesh color distribution in BC1P2 population (P2 is the white flesh parent PI183967). Figure S3. Violin and box plots depicting phenotypic distribution of flesh color, flesh extract color and flesh chlorophyll content in SY2017W experiment. a Violin and box plots depicting flesh color. b Violin and box plots depicting flesh extract color. c Violin and box plots depicting flesh chlorophyll content. Figure S4. Linkage map constructed using a 234 F2 population in SY2016W experiment. Figure S5. Linkage map constructed using a 125 F2 population in BJ2017F experiment. Table S1. Summary of populations used for phenotypic data collection, QTL mapping and GWAS analysis. Table S2. Statistics of two linkage maps. Table S3. Information of markers mapped with 234 9110Gt×PI183967 F2 population. Table S4. Information of markers mapped with 125 9110Gt×PI183967 F2 population. Table S5. Information of the CG lines used in this study. (PDF 663 kb) [file 12870_2019_1835_MOESM1_ESM.pdf]

Supplementary Fig. 1

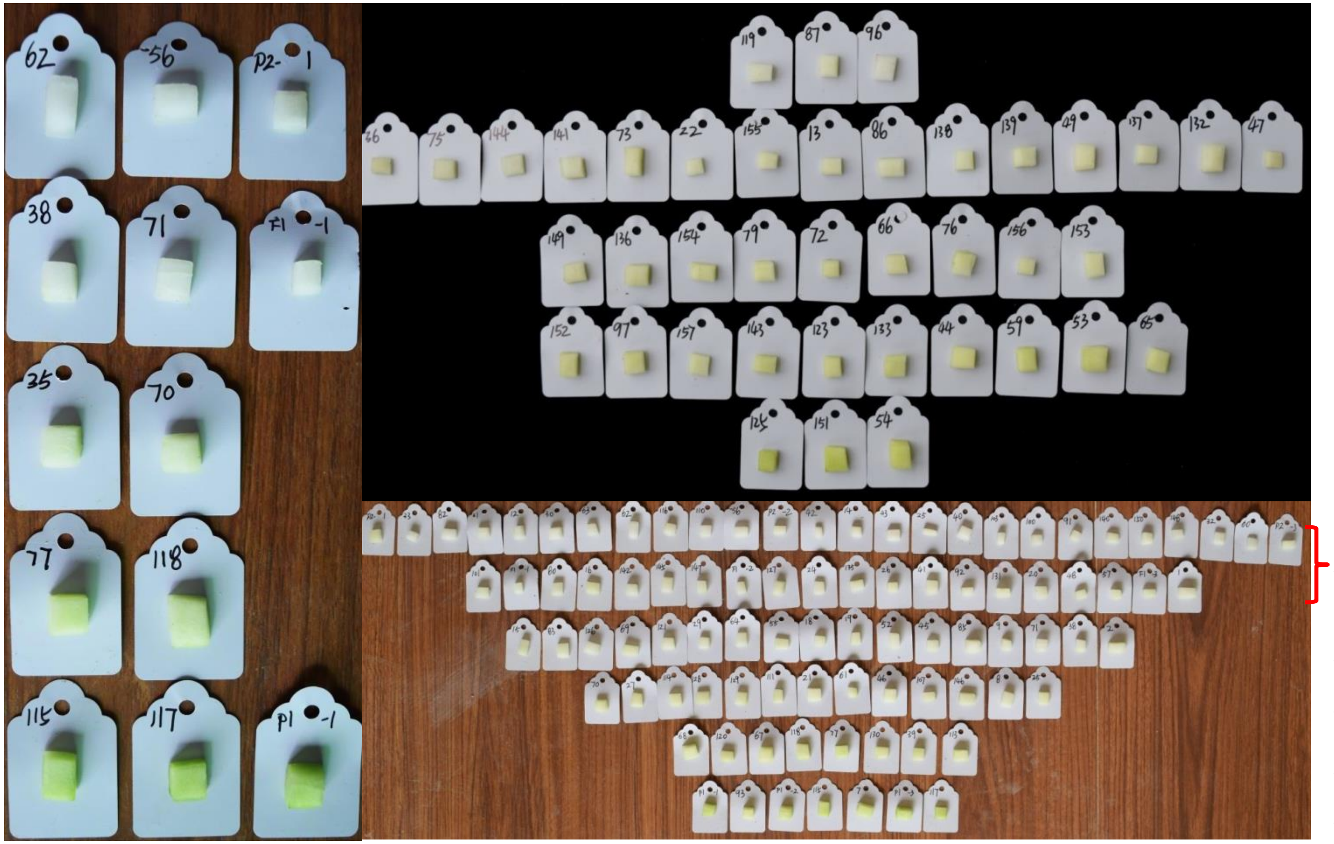

Supplementary Fig. 2

BC1

A

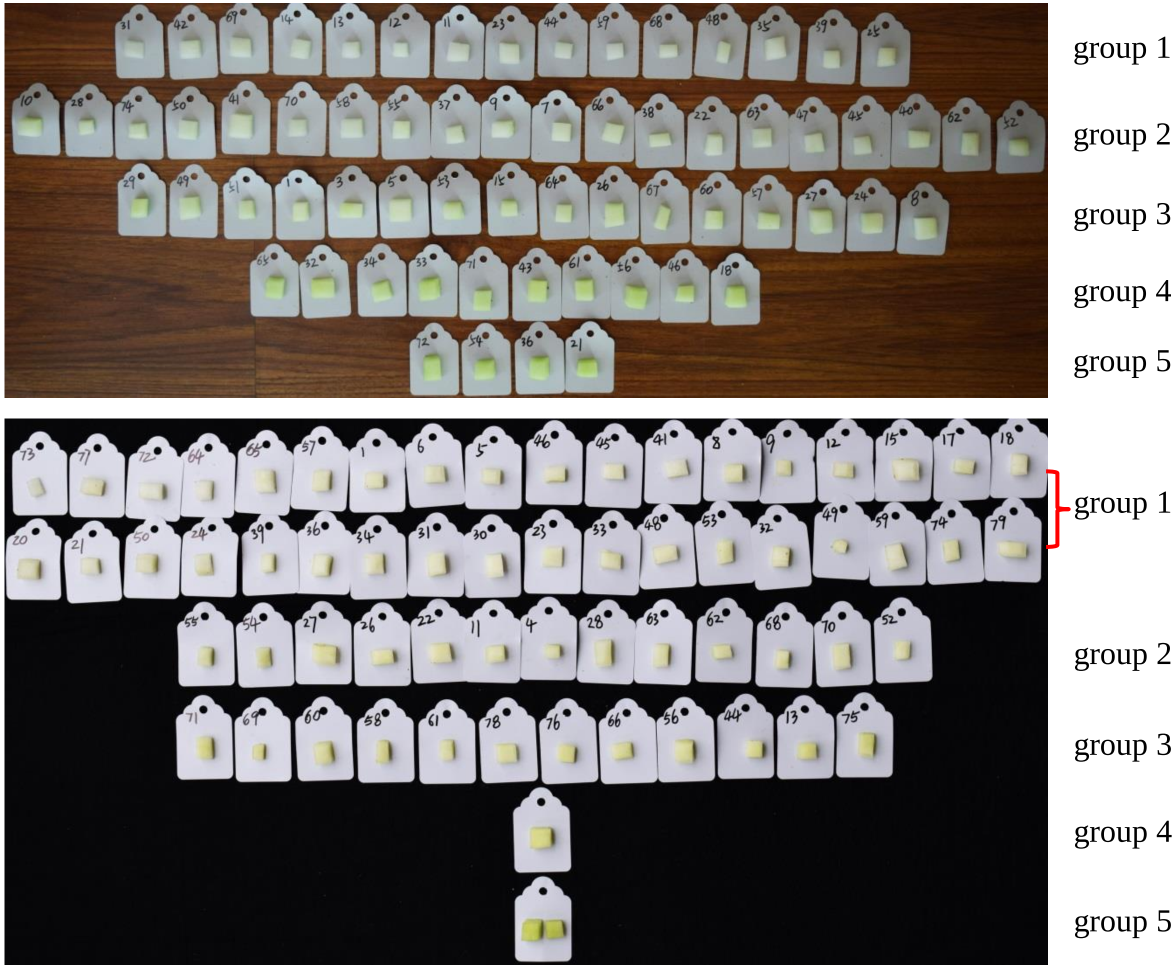

group 1

group 2

group 3

group 4

group 5

B

group 1

group 2

group 3

group 4

group 5

BC2

Supplementary Fig. 3

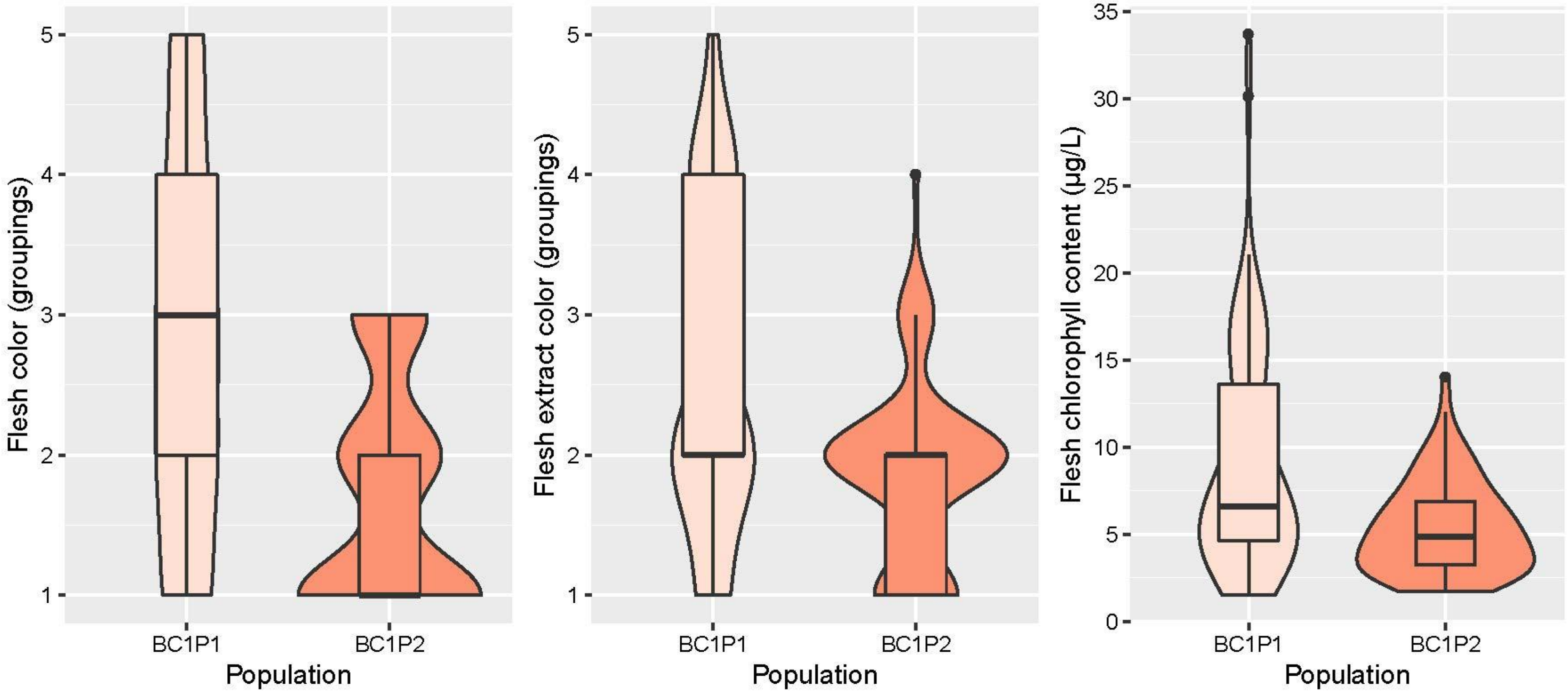

Supplementary Fig. 4

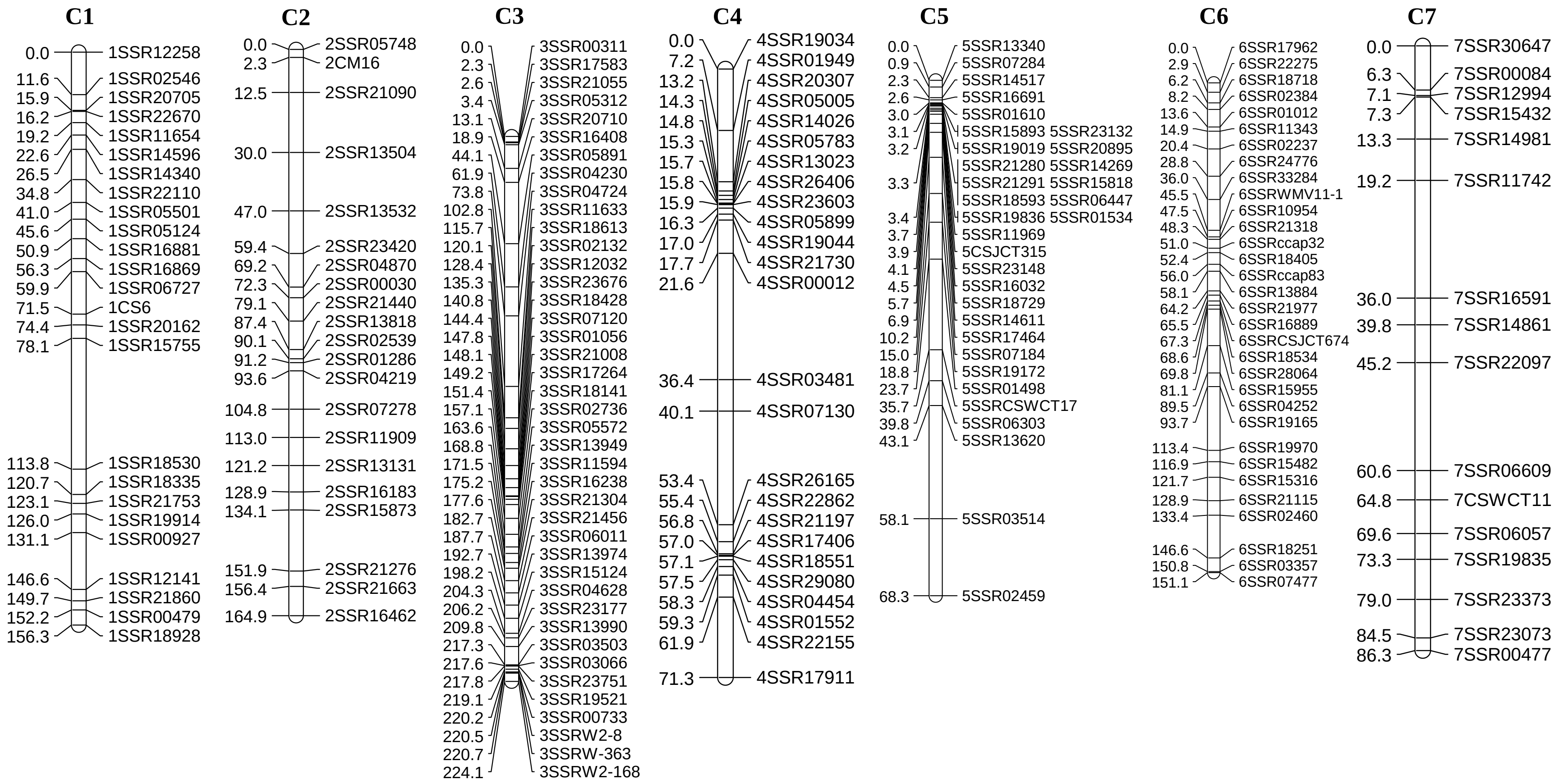

Supplementary Fig. 5

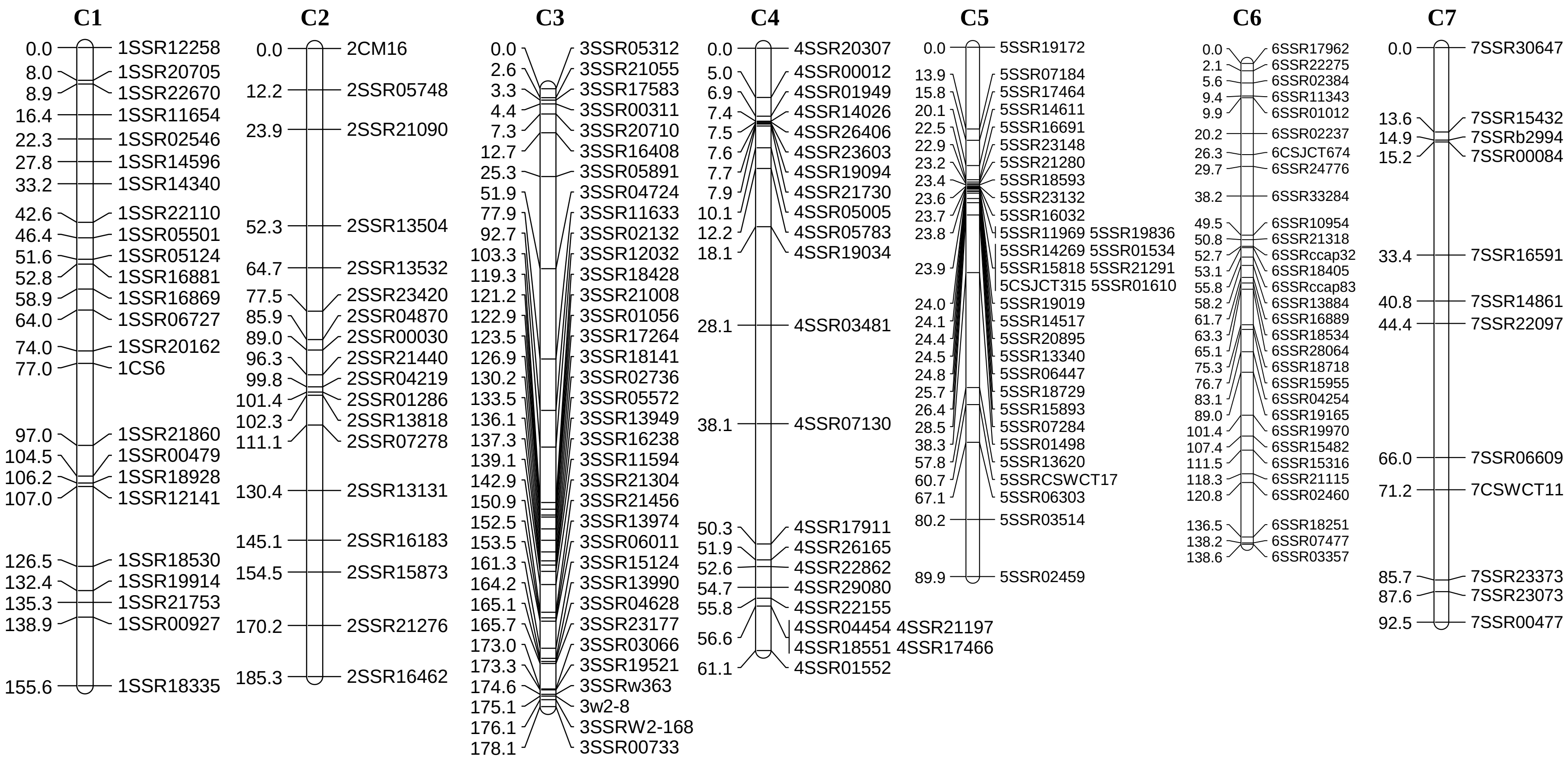

Table S1. Summary of populations used for phenotypic data collection, QTL mapping and GWAS analysis

Populations[a]	Experiments	Location	Year	Season and environments	Experiment design [b]
$234F_2$	SY2016W	Sanya, Hainan, China	2016	Winter, screen house	234 individuals
$125F_2$	BJ2017F	Nankou, Beijing, China	2017	Fall, plastic house	125 individuals
$140F_2$	SY2017W	Sanya, Hainan, China	2017	Winter, screen house	140 individuals
$78BC_1P_1$	BC_1P_1	Sanya, Hainan, China	2017	Winter, screen house	78 individuals
$77BC_1P_2$	BC_1P_2	Sanya, Hainan, China	2017	Winter, screen house	77 individuals
CG	CG2017S	Nankou, Beijing, China	2017	Spring, plastic house	115 lines, 3 reps, 5 plants/rep, CRBD
	CG2017F	Nankou, Beijing, China	2017	Fall, plastic house	115 lines, 3 reps, 5 plants/rep, CRBD

[a] $234F_2$: 234 F_2 individuals, $125F_2$: 125 F_2 individuals, $140F_2$: 140 F_2 individuals

$78BC_1P_1$: 78 BC_1P_1 individuals, $77BC_1P_2$: 77 BC_1P_2 individuals

[b] CRBD: completely randomized block design

Table S2. Statistics of two linkage maps

$234F_2$	Chromosome	Map Length (cM)	#Loci Mapped	Map Interval (cM)
	1	156.3	25	6.25
	2	164.9	21	7.85
	3	224.1	41	5.47
	4	71.3	25	2.85
	5	68.3	32	2.13
	6	151.1	32	4.72
	7	86.3	16	5.39
	Total	922.3	192	4.95

$125F_2$	Chromosome	Map Length (cM)	#Loci Mapped	Map Interval (cM)
	1	155.6	24	6.48
	2	185.3	18	10.29
	3	178.1	35	5.09
	4	61.1	23	2.66
	5	89.9	32	2.81
	6	138.6	30	4.62
	7	92.5	12	7.71
	Total	901.1	174	5.67

Table S3. Information of markers mapped with 234 9110Gt×PI183967 F_2 population

Chr	Loci	cM	9930 V2 position	Left primer 5' to 3'	Right primer 5' to 3'
Chr1	SSR12258	0	467457	GGTTCAAATTTCAAAGTGTATGAAT	GCCAAAGCTTCATAGTGAGCA
Chr1	SSR02546	11.637	3445531	CTTCGCCTTTCCTATCCTCC	CAAACCTGATAAGTGGATTGGG
Chr1	SSR20705	15.857	2135846	CCTTTCCTTACCCATCCCAT	ACCCATTTGAATCAGCTTCG
Chr1	SSR22670	16.215	2310326	TTCTCGATTGGTTAGTCGGG	CCATACCTTTGTGTTCCATCAA
Chr1	SSR11654	19.221	2967958	AGACCCTTTCCAGGAACCAT	CAGAGGTGTCTAAGCTCCCG
Chr1	SSR14596	22.62	3951558	TTTGGCTGTGATGATTCTGG	TGGGAAACATCGTCAAAATTA
Chr1	SSR14340	26.457	4613114	TGACCGGAAGTATCATTGGAG	TCCTTTCCTTGTGAATGGAAT
Chr1	SSR22110	34.75	5270783	TTCCCCATTCTACCCCCTAC	GGGAAAAACAAAATGGATGAAA
Chr1	SSR05501	41.018	6038250	GGAATTATGCAATTTGGGCA	AACCAAACAATGCCCTAGCA
Chr1	SSR05124	45.617	6683318	TCTTTACCAATTTTATGGTGATGTT	AATCAAGGGTGCAAATGTCA
Chr1	SSR16881	50.939	6858338	CCCTCTCAACATTTTCCACAA	CGAGGAGACTTGATGGGATG
Chr1	SSR16869	56.336	7463806	GAAAACATGAAGGCCGTTGT	CCATTTGTCAGTGCCTTTCA
Chr1	SSR06727	59.886	8096485	TCGCCTCAATCACAAAATGA	CTGCCATTGTTTTTCACTGC
Chr1	CS6	71.453	13170009	GGAGGAGGTACATGGCTTTG	TGAATCTGATCTTTCTACCTTCACA
Chr1	SSR20162	74.437	12386750	TGCCTTCCCATATCAAAACA	GGTCATTGTCAGGAAACAAAAA
Chr1	SSR15755	78.141	12462918	GTGCCTCAGTCGGTTTTGTT	GGGAGGGGAAGGATTCAATA
Chr1	SSR18530	113.806	18981201	CCACAGTCCCACACACAAAG	GGCGTTTTGTGAAGACAGATT
Chr1	SSR18335	120.745	20251684	CACCGATTTGAGCACCTACA	TGTCAATACAGGCAACACTCAA
Chr1	SSR21753	123.059	20714208	GGGTGAAATGGTAATTTTGGG	TGTGTTCAACGGGTGAGAAA
Chr1	SSR19914	126.007	21334829	ATGGTCCACCAAACAAATGG	GCTGTACTTGGAATCACTTCCC
Chr1	SSR00927	131.147	20821600	CTgCTgAAAAggTgTTggCT	TggAAATgATCTAgCTgCCA
Chr1	SSR12141	146.574	26083906	AAGCATCAGTCTCTTTTCATTTCA	TGCAACAAAGAAAACAGCATC
Chr1	SSR21860	149.695	27230895	GAAGTACACATTTGAATCTTCTTTTCA	GCTTCCAAAATTGAATTGTCC
Chr1	SSR00479	152.151	7986587	GGACGCCACGATTCTACAAG	GGATGTTCGAGTTGCAGACC
Chr1	SSR18928	156.336	26541128	TTCAAAATGTAGAGTTTTCGGACA	CGTGAATGAATTTGTAACCTTTC
Chr2	SSR05748	0	89863	TGTGGCCTGTGCTAAAATGA	TTTGGAAAAGCTAAAGCCCA
Chr2	CM16	2.325	487540	TGCCTGTTGTGATTGAGGAG	TTCTTCTTACCTCCGCCAAA
Chr2	SSR21090	12.456	1858848	CTGGAAATGGGGTTGGATTA	TGAATCAGCCGTTTCTGTGA
Chr2	SSR13504	30.012	4372184	CATCCCTTTTCCCTTCTTCTTT	TCCATGTGGAAGAATAACCCA
Chr2	SSR13532	47.041	5647445	AAACCCAAGAATTGTAAACCCA	TGATCCATTTCTATTCCTAACATTGA
Chr2	SSR23420	59.409	7873772	GAAGGGATGGTAGATGAAGGG	CTTCTCCCCTTTCTTGGCTT
Chr2	SSR04870	69.235	10118535	TACATGCCCCCTTGAAGAAG	TTCTTGGAGGATTTGCAGAAA
Chr2	SSR00030	72.291	10670350	TGAAATTGCTTACCCTTTGACC	CCATGTTTTGTAGGGATCGAG
Chr2	SSR21440	79.127	12743133	TTGTATCAAATTGTGCCTTAAAAGTT	TGCATGCTTATCTCCTTTTCA
Chr2	SSR13818	87.441	14974382	TTGTTAGTTCATTTGAGGTGTCAAG	TCCATATTAACTCTCTCAGGCTAACA
Chr2	SSR02539	90.083	13768896	AAAAATGATCAGCTCGATGAAA	GCAAGCGCTTTCCAATCTAT
Chr2	SSR01286	91.192	14658378	CCGAAAACCATTGTTCAAGC	TTTAGCTTAGTTTCCAAGCACTGA
Chr2	SSR04219	93.595	13713498	GAGACATTGTGGGCATTTGA	CTCATTTTCATCCAAAGGGC
Chr2	SSR07278	104.848	17016846	ATCGCCATGGATTTCATCTC	CCACTCTTCGAGTACCGCTC
Chr2	SSR11909	112.955	17471678	AATAATACCAGTGGCCCCATC	AAAGCTCCCTCCTCCCCTAC
Chr2	SSR13131	121.188	18505180	AAAGCAGAGTATGGCATGGG	AAAAGCCAAAGAACCCAACA
Chr2	SSR16183	128.885	No Hits	GGAGAAATTTGATGGTGTAGCC	TTGCAAATCTCTAATACTTTGCCTT
Chr2	SSR15873	134.062	19269078	GCCAAAAATCCGAGAACAGA	TCCTGGGATTTTGATTTGCT
Chr2	SSR21276	151.869	21047286	TCGATGATAAATCCATCACATTTT	CGCTCTTGCCTATCTTGCTT
Chr2	SSR21663	156.439	21368763	ATCCCCATAATCCCATTTCC	AGCATTATGAATGGAGGGCA
Chr2	SSR16462	164.853	22621989	TCACCTTCATGATTTTGCCA	GTCTTCAACGCAACCCAAGT
Chr3	SSR00311	0	No Hits	TGAGGCAGAATCGAGAGTAGG	TGCAAAACTTCTTCTCGAATACA
Chr3	SSR17583	2.26	No Hits	GTTGATAAAAGAAGCCGCCA	TCTAAGCCGCCCTTAAAACA
Chr3	SSR21055	2.622	No Hits	CATCCTCATCCACCCAATCT	TCCAAACAAGTCTGACAAGGAA
Chr3	SSR05312	3.432	2368898	GCCATGGAAAAGGTTTGAGT	TCAATTATGCCCCTTATCCC
Chr3	SSR20710	13.05	3610715	TAGAAGAAGCATCGGCAGGT	GAACTGGGGGTTTACCAGAA
Chr3	SSR16408	18.877	5630730	GTTGAATTATTGGAGGGATTTTT	CATTAATTTATTCAACCGTTTGGT
Chr3	SSR05891	44.129	No Hits	GTTTGGGTATAGGGAGACCG	TGAGATGTCGAGAACTCCATACA
Chr3	SSR04230	61.894	9944424	CCGTACGATCCTTCCACATT	CAAGGAGGACCCATCATTGT
Chr3	SSR04724	73.824	11217547	CTTTTCGCATTTCTTGACCA	CCACATTTCTAAGACCATGGTAGA
Chr3	SSR11633	102.779	15920930	AATGAGATAACATATGGATTTGTATGA	CACCGAACTGAAGTGTGCAG
Chr3	SSR18613	115.653	19918371	CCACATAAAACAGCAAGGCA	TGTTGGTTGGTTGACTAGCATT
Chr3	SSR02132	120.068	20792964	CAATTGGTATGAGTGAAAGATAAGC	CTCTGGTCCACCCAATCCT

Chr3	SSR12032	128.383	23296186	GGGGGTTTGAATTGAATGTG	CTCTTCTTTCCCCCTTTGCT
Chr3	SSR23676	135.326	25442405	TCCACTTTTGTCCTCTCCTCA	CAAAGTAATAGTGTGCTGTGAAAGA
Chr3	SSR18428	140.772	26417833	CCATTCACTTCCTTTCCAGC	TGGTTTCAAGACCACCCTCT
Chr3	SSR07120	144.442	No Hits	GATCAAAAGATCCAACTAATAACCA	TCGTCCAAATAGTTGTTCTTACCA
Chr3	SSR01056	147.762	No Hits	AAAgggAAAggTAAATTgCCA	AgCAgTTCggATgATATTggA
Chr3	SSR21008	148.091	No Hits	TTGTCCACCTTGGATGTGAA	CCCACTTTTCCCTTTAACCC
Chr3	SSR17264	149.177	27811206	CCCTCCCACCTTCACTATCA	ATTGTCAACAAGAAACAGTGTGA
Chr3	SSR18141	151.421	28666375	ATGTCAATATGTGGGACCCG	TCGTAATAAATGGATGGCCC
Chr3	SSR02736	157.142	No Hits	ATGGGTGTGTTAGAAAGATTAGGA	CCAATTGATTTGGATTGGACA
Chr3	SSR05572	163.639	No Hits	GCAAACCATAAGTTTCCCCA	GATCGATATTGCAACGAATTACA
Chr3	SSR13949	168.822	No Hits	CCCATCAATCCCAGTTCATC	GTGCTGTTGCCATAAACCCT
Chr3	SSR11594	171.48	31068516	GGCGAAAGATGAACAGAAGC	CCATGTTTTCGGTTTCCTTC
Chr3	SSR16238	175.182	31163143	TCCGGATCTCTACCAAATGAA	TCAGAGCATAAAACCAGTGTGT
Chr3	SSR21304	177.558	No Hits	CCTCACCATGGTATTATCAATCG	GAATTCTTCTCGAATGTTTCCAA
Chr3	SSR21456	182.67	No Hits	AAGTGGGAAGGTTTGGAGGT	TTCCTTTTTCCCCCTTCATT
Chr3	SSR06011	187.736	No Hits	AGGGTCCAACTCAAACTCAAA	GCCCACCCTTAACATCTTACC
Chr3	SSR13974	192.712	32586332	ATGGAGTGTCAAGCAAGCAA	ATTGGGATTAGCAGCTTCCC
Chr3	SSR15124	198.227	No Hits	TCCACCCAATCTTTCCACTC	AGTGCTCTTCACATTTGCCA
Chr3	SSR04628	204.331	No Hits	CCAACTTTTCGTAATGATTGAGAG	GTGAGCGTCAGAGCAATTCA
Chr3	SSR23177	206.199	No Hits	TGGATGAATGATGCCACAGT	CAAAAGCCTGTCTTGGTAAAAA
Chr3	SSR13990	209.772	No Hits	CCAAATGCGGTGAAAAAGAT	GGGATTCTCTCTACCCCCAA
Chr3	SSR03503	217.261	No Hits	AACAGGCCTCAGAAGCAATG	CCCAGGTTTGAGTTCCATCA
Chr3	SSR03066	217.631	37239584	CAAAACTTAAGGACCGAAAGGA	ACATGGTTGGTTAGTGGCCT
Chr3	SSR23751	217.842	37093817	CATCCCCATCCCTTTCTTTT	ACGTGGAGGGATAAACGTGA
Chr3	SSR19521	219.138	No Hits	CAACCACACACAAATTCCCA	ATATGCCGCCCTAGAAAACA
Chr3	SSR00733	220.231	No Hits	TCAAATTGTGATAGAGAGGCAGA	CCTCACAACCAAAAGGTCAAA
Chr3	SSRW2-8	220.47	No Hits	gAACCCAAATTggCATTAggT	gCATgTCTCTCTCCCTCAgC
Chr3	SSRW-363	220.652	38975987	TGCCTTCTTCCTTTCCTTCA	CCAAACAACACCATCAGAAACA
Chr3	SSRW2-168	224.093	39694820	TGTCCTTCCCATTCAATCGT	CCACAGTTTGTGCATCCAAG
Chr4	SSR19034	0	2972019	TCTTGCTTATCCCACCGTTC	GGGTTTCAAAACATTAACCCA
Chr4	SSR01949	7.249	823261	AGGAAAACCGGAAGCAGAAT	TCCACAGAACAACCGTGAAA
Chr4	SSR20307	13.197	No Hits	CCAAAAGGTCATTCGGTTCA	CGAAAAGTCAAGAAGAAACACAA
Chr4	SSR05005	14.252	10542520	CAGATTTAGCCAGTGGGTCC	ATCAGGGATCAGTCAATGGC
Chr4	SSR14026	14.799	No Hits	TACCGGAGAAATCATCGAGC	TCGCTAAACTCCAACACGAA
Chr4	SSR05783	15.304	3363484	AAAAGTCCCACAACATATGGAAA	TGGACAAACCAAGTCCCACT
Chr4	SSR13023	15.714	4471539	GTGGGGAGTGGCATATTCAG	AACCCGGCAGGATAATTTTT
Chr4	SSR26406	15.757	5276352	AGCACACTTGGGTGGATTTC	CCTTTTCATTTGCAAACTTCC
Chr4	SSR23603	15.89	5973993	GACCATTTACCTTGCCATTTT	GAAAGCGAGCTAGGGCTTTT
Chr4	SSR05899	16.285	4790192	TAAGAGCAAAAATCCCACGC	AGCTCAATCAACGTCAAGAGAA
Chr4	SSR19044	17.041	No hits	TgAAAAgAACATATggggCA	TgCAAACATAACAATTCgATCC
Chr4	SSR21730	17.669	No Hits	TTTTGTGCTGCTGATGAAGC	TTCTCATCGCAGGTGAAGTG
Chr4	SSR00012	21.63	No Hits	TCTCACCATGGTCACCTAATG	GGTCATTGAAGAGTCAAGTTGG
Chr4	SSR03481	36.426	12971131	TGTCTGTCCTTTTCCCTCCTT	AGAAACATGGTATGATATGTTGGA
Chr4	SSR07130	40.142	No Hits	CCACACACACACACAGTCACA	TCCCATTGTCCCTCACTCTC
Chr4	SSR26165	53.448	No Hits	TCTTTTGTTGGTGAAAATTGAAA	CCTTCTCATGTGTATTGTCTTTTG
Chr4	SSR22862	55.425	No hits	AATTGGGTCAAAGAAAGGGG	TCGTCAAAATGAATCTCCGA
Chr4	SSR21197	56.801	23134766	AAACCATTTGGTATTGGCGA	GTCCAACCAAACCAAGCCTA
Chr4	SSR17406	56.997	No hits	GAGCCATCCATCAGAGAGAGA	ACCCACAAGCTTCAGAGGTC
Chr4	SSR18551	57.103	21268560	GATGTGCATGTGATCCAACAG	TGAATCTACTTGGGTTCGTTGTT
Chr4	SSR29080	57.513	No Hits	TTTTGCAATCAACCATTTTCC	GCCCATTCCCAATATATCCC
Chr4	SSR04454	58.328	16371689	GGAAAGTGTTGTGTTGCTCTTG	GATCCATTTGATGCATTGCT
Chr4	SSR01552	59.269	No hits	CACGACTTGCCATTAACCCT	CCCAAACTTTCCCCTCAAAT
Chr4	SSR22155	61.935	22948132	AAACGGACATGTTAGGTGGC	TGGATTATTTAGGACAAACTGCAA
Chr4	SSR17911	71.265	No hits	TGGAAGAGTGAGAAATGGGC	TGGGAGAAACGTTCTTTATTTTT
Chr5	SSR13340	0	No hits	GCTTTACATGGCTTCTCCCA	TTTTCCTTGGCGACGATTAG
Chr5	SSR07284	0.87	No hits	CTGCAGAATGTTTTGAGGCA	CCAACCTTGGAGGTGTTGAT
Chr5	SSR14517	2.276	9375222	TCCCCATATGTTCTTCTCGC	AAGCTAAGGCAAACCAACCA
Chr5	SSR16691	2.604	9141668	CATGTTGAGTGGAAAAATTGAAA	TTCGTACATTGTCAAATGCG
Chr5	SSR01610	2.979	No hits	TGGTTGTATATAGAAGGAAGATGTTGA	CCCTTTGAACGAAATCCAAA
Chr5	SSR15893	3.09	No hits	ATGGGAGCTACACGTTGAGG	ATAGGCTTCCATTCCAGGGT
Chr5	SSR23132	3.149	15531345	GCCCTATCACTTCTTTGTTTTCA	CAAGAAATGGGGTGGAAGAA

Chr5	SSR19019	3.214	No hits	AgCTAgCTgCACCCACAACT	AAAgTATCCggAATCTTgTCAATC
Chr5	SSR20895	3.218	No hits	CCTCTTCCTCCTCTTCCTCC	CCTTCCCCAATGTAGCAAAG
Chr5	SSR21280	3.253	7486163	CCATGATTTCAAATACCCCTT	TCTGTTTTCAAAATTGTTCGGA
Chr5	SSR14269	3.27	13002103	GGAAATGGGAGATACACGGA	GCACTTTCCAGTTCCATGCT
Chr5	SSR21291	3.273	16273295	CACTCCACAAGCAAACCAAA	CTATGGCGGAGAAGGAGTTG
Chr5	SSR15818	3.281	18966224	GGACATGTCAACTCCCCTGT	GCCTCTAGCCTGAAAGACCA
Chr5	SSR18593	3.321	129876	TGGGCCTCAATCCTTGTCTA	ATAAGGTGTGTGGGCCAAAG
Chr5	SSR06447	3.332	8580058	AAGTATGACGACACCCTTCG	CGCAAAACCGAAAGGTACATA
Chr5	SSR19836	3.419	No hits	TGCAGAAACATGAAAAGGGA	TGATTGAGTCCCATATGCCC
Chr5	SSR01534	3.446	No hits	CTTCTGCTTCCGCTTCCTTA	GGAAGAAACCCACCAGAACA
Chr5	SSR11969	3.673	5973902	GGGTCATACCCAAAAGGGAG	TGCTTTAGCCGACAACTCAA
Chr5	CSJCT315	3.922	No hits	CCACgAAATACAgATCAgCAAC	CACgTTACATTggACgAgAgAT
Chr5	SSR23148	4.081	16426691	TGCCGTTTGGATCACATAGA	CAATACGCACAAAAAGCGAA
Chr5	SSR16032	4.512	No hits	TCATGTCATCAATCACACCTTTC	TTTTTATCTTTTGTCGAGACTTGTTTT
Chr5	SSR18729	5.708	13006047	TCCAATCCCACTCTGGTTTC	TCTTTCCAACACTAACCTTGGATT
Chr5	SSR14611	6.886	No hits	ATTTGGCTTCCAGGTTGATG	GAATCGAAAACCGATGAGGA
Chr5	SSR17464	10.205	21187949	ATCTCAGCCCTTGGATCCTT	TCTTCGAGAAATGGGATTTTG
Chr5	SSR07184	15	No hits	CACCATTTTCCACAAAACGA	GCCATACCCTTCAACATAGGA
Chr5	SSR19172	18.828	22085961	CGAGGAGAAGGACTACGACG	CGTGTGGAGCTTTCTCACAA
Chr5	SSR01498	23.738	22655145	GGCGCCACAAATATTCAACA	CCACAAACGTAAAGAGATTCACA
Chr5	SSRCSWCT	35.735	No hits	TTgAATTATgggTTCATTTTT	gACAATgATAAACTTCCCTgA
Chr5	SSR06303	39.796	24878262	AGCTCTCAACAACGAAGGGA	TGACTTTCTTGATGGTACCGC
Chr5	SSR13620	43.083	24082650	TTTTCCGGTTCTACAATGGC	AGTTCAATCTTGTATGAGTGATATTGA
Chr5	SSR03514	58.057	26718521	TAGGGTCCCCTTCCCTCATA	GGGTACCCAAAAGCAAGTGA
Chr5	SSR02459	68.342	27816197	TCGGAAGATGGGTTATTTGG	TGACCCCTCACATTCTCTCC
Chr6	SSR17962	0	776867	CAAAAGAAAAGCGCAAAAGG	GTCCCACTCCCAAAAACAGA
Chr6	SSR22275	2.884	1016060	ATACTTCGCCATTGACGCTT	TTACTAGGCGTTGTGGGAGC
Chr6	SSR18718	6.173	1611048	TGAAGCAAAGTAACCCCACA	CACAAATGGATTACAGAGCGAA
Chr6	SSR02384	8.238	2783756	AAAAATCCGACAAATCGTGC	GGTCAAATGTTGCCTTTTGC
Chr6	SSR01012	13.621	3855950	TCCAAAAATCGCGACCTAAA	GTGAGCCGTTGATTTCTCGT
Chr6	SSR11343	14.943	4133077	GTGGGGTTGCTTTTGGATAA	CAATGGTTGCTTTGCTTCAA
Chr6	SSR02237	20.389	5124994	CTGAAAACTGGGGAATGGAA	GTTTGTTGCCTCGAAAAGGA
Chr6	SSR24776	28.82	5812826	GTTCCTTGGTGCCATTGTTT	TCCAATTCCCTCTCTCTTCG
Chr6	SSR33284	35.975	6610818	TGGGAAATGGATAATTTGGTG	GGCATCCATCATTTCTTTGG
Chr6	SSRWMV11	45.543	9025951	ACAAAGCTTCTCCGCAAATG	GGAGGGAAAGGAAGGAGAGA
Chr6	SSR10954	47.503	9453905	TGCAAAACCAATTATTTGATATAGAGA	TTTCGGCAAAAGAACTAGGAG
Chr6	SSR21318	48.268	9372844	GACACCCCATTCCTCATCAT	GCTTCATCACTCCCAATTCAG
Chr6	SSRccap32	50.968	9764418	CGATCGTATTGATTAGATAAGCG	CGATTCGTCACTTTTCTTTCTTC
Chr6	SSR18405	52.359	10051003	CGCAGGTGCATCTCATGTAA	GACAAACAAGGGGACGAAAA
Chr6	SSRccap83	55.989	No hits	CgTgAACCTCCgATCTCATT	AgTCAAAgTTggCCAgTCgT
Chr6	SSR13884	58.08	11069339	GGATAATCCTGATTCCTGTGG	TGTGCAACTGAAAACGAAGC
Chr6	SSR21977	64.185	No hits	ggTgTACCgCATAATCCACA	TTCATCCAACTTTTATTgCATCT
Chr6	SSR16889	65.47	14857891	TCACATATTGGAATTTGAGGACA	AAGAGCAAACATTTGTTGAAACTC
Chr6	SSRCSJCT6	67.291	No hits	TAgAAAggAAgggATgTgATTAgg	ACAggTggTTAgAggTTAgAgCTg
Chr6	SSR18534	68.611	16606287	GCCCTGAAACTGAAGCTGAG	CTGATTGCCAAAGTCAAGCC
Chr6	SSR28064	69.752	16465180	GAAATTAATTAAGATCAAGAAAGGGA	ATCACTTGCATCACGTCTGG
Chr6	SSR15955	81.11	18995075	TTTGAGCCTTGAGGCAAAGT	GCAATTCAACGTAATGGGCT
Chr6	SSR04252	89.507	20075416	AAAGAACACACATGGTGGTGG	AAGGAGTGTTTGAATAGGCCG
Chr6	SSR19165	93.738	21344610	AATCCACGTTGGTTGTCGTT	GAAGGGCCAAAAATGTTTCA
Chr6	SSR19970	113.357	23339816	TACCCGGTAATCTTCCCCTC	CAACAGAAGCAAAGTAGGTGGA
Chr6	SSR15482	116.944	24003956	AAAAACAGCAGGGCAGAAGA	GTTGAGCTGTCTATGGGGGA
Chr6	SSR15316	121.667	24577348	TCCAATTTTCTGGCGAAGAC	CAACCGAAACAACGTCTCCT
Chr6	SSR21115	128.901	25689547	GCAGCAGTCACCCAACCTAT	CACTGTGAACATATGGGAAGGA
Chr6	SSR02460	133.443	26252541	CTCAGAAACCCTTCCACCAA	CTGTACCGCGAGGACAGTTT
Chr6	SSR18251	146.586	28192598	AAATGGCAAAATAATGCATGG	GAGACAGCCACAGAGATTTGG
Chr6	SSR03357	150.759	28813523	AAAAGGGCAAGTCAAAACCC	GGGGAGGAAGAGAGACCCTT
Chr6	SSR07477	151.115	29009111	CGTTTCATTCATTGCTGCAC	GCCCAGATTTGGTCATTTCTT
Chr7	SSR30647	0	448536	GAAATGAGAGCAAGTTGCAAAA	AGGCGTAAATCTGACCGTTG
Chr7	SSR00084	6.298	40139	AAGTGTCAGCATGTCGTTCG	AAACGTCAGCCACACCTTTC
Chr7	SSR12994	7.084	2869178	TCATCCTGCAATGCAAAGAC	ACCGTGGATCTTAAGCCAGA
Chr7	SSR15432	7.275	4372675	TGAGAATTGGATGTGCGAAA	TTAGTTCATTGACATGGGATGAT

Chr7	SSR14981	13.311	6577201	GAGTGCTTGGAGTTGGGTGT	GAGTGGGAATGAGGAGGTGA
Chr7	SSR11742	19.19	9462250	GCTATCCCCAAGGATGATGA	AGCTTGGCTTCGTCTTTTGA
Chr7	SSR16591	36.039	12291486	TTTTGGGTAGAAATCAAGAAACG	GGGAATCAAACATTCATGGC
Chr7	SSR14861	39.756	12975963	ATTTCTTCCCCCACCAAAAC	ATGAATCCTCCTCCCAGAGC
Chr7	SSR22097	45.171	13483521	ACGGTCATCCGAATTCTCAG	CAACAAACGATCCAACATCG
Chr7	SSR06609	60.601	15169712	TCTAATGGAAACCCTCTCCG	GAGACGGAATCGGAATCAAA
Chr7	CSWCT11	64.823	No hits	ATAGGCAATATGGCTTCT	CACTTCAATGGAGTTTCG
Chr7	SSR06057	69.647	16098193	GTTCGTGACTCACCAGACCC	TCCTGAATTTTGGGAATTGG
Chr7	SSR19835	73.286	16698722	CAAGCCTTGAATGGTTAAATACA	AAAGACTTGGTTGATCACTTTCA
Chr7	SSR23373	78.983	17467963	CCCACCATGCAAAACTCTTT	GTCTTTGCGCACCTCTCACT
Chr7	SSR23073	84.494	18153573	ATGAGGGAAGACTTTGGGGT	TTTCGTGAAGAAGGGGAAGA
Chr7	SSR00477	86.271	19197072	TATTGCGATGGTTTGACGTG	GCAAATTCCGGAGTTCGTTA

Table S4. Information of markers mapped with 125 9110Gt×PI183967 F_2 population

Chr	Loci	cM	9930 V2 position	Left primer 5' to 3'	Right primer 5' to 3'
Chr1	SSR12258	0	467457	GGTTCAAATTTCAAAGTGTATGAAT	GCCAAAGCTTCATAGTGAGCA
Chr1	SSR20705	7.998	2135846	CCTTTCCTTACCCATCCCAT	ACCCATTTGAATCAGCTTCG
Chr1	SSR22670	8.91	2310326	TTCTCGATTGGTTAGTCGGG	CCATACCTTTGTGTTCCATCAA
Chr1	SSR11654	16.356	2967958	AGACCCTTTCCAGGAACCAT	CAGAGGTGTCTAAGCTCCCG
Chr1	SSR02546	22.315	3445531	CTTCGCCTTTCCTATCCTCC	CAAACCTGATAAGTGGATTGGG
Chr1	SSR14596	27.786	3951558	TTTGGCTGTGATGATTCTGG	TGGGAAACATCGTCAAAATTA
Chr1	SSR14340	33.152	4613114	TGACCGGAAGTATCATTGGAG	TCCTTTCCTTGTGAATGGAAT
Chr1	SSR22110	42.641	5270783	TTCCCCATTCTACCCCCTAC	GGGAAAAACAAAATGGATGAAA
Chr1	SSR05501	46.399	6038250	GGAATTATGCAATTTGGGCA	AACCAAACAATGCCCTAGCA
Chr1	SSR05124	51.559	6683318	TCTTTACCAATTTTATGGTGATGTT	AATCAAGGGTGCAAATGTCA
Chr1	SSR16881	52.754	6858338	CCCTCTCAACATTTTCCACAA	CGAGGAGACTTGATGGGATG
Chr1	SSR16869	58.936	7463806	GAAAACATGAAGGCCGTTGT	CCATTTGTCAGTGCCTTTCA
Chr1	SSR06727	64.009	8096485	TCGCCTCAATCACAAAATGA	CTGCCATTGTTTTTCACTGC
Chr1	SSR20162	74.037	12386750	TGCCTTCCCATATCAAAACA	GGTCATTGTCAGGAAACAAAAA
Chr1	CS6	77.003	13170009	GGAGGAGGTACATGGCTTTG	TGAATCTGATCTTTCTACCTTCACA
Chr1	SSR21860	96.979	27230895	GAAGTACACATTTGAATCTTCTTTTCA	GCTTCCAAAATTGAATTGTCC
Chr1	SSR00479	104.505	7986587	GGACGCCACGATTCTACAAG	GGATGTTCGAGTTGCAGACC
Chr1	SSR18928	106.244	26541128	TTCAAAATGTAGAGTTTTCGGACA	CGTGAATGAATTTGTAACCTTTC
Chr1	SSR12141	107.021	26083906	AAGCATCAGTCTCTTTTCATTTCA	TGCAACAAAGAAAACAGCATC
Chr1	SSR18530	126.499	18981201	CCACAGTCCCACACACAAAG	GGCGTTTTGTGAAGACAGATT
Chr1	SSR19914	132.445	21334829	ATGGTCCACCAAACAAATGG	GCTGTACTTGGAATCACTTCCC
Chr1	SSR21753	135.303	20714208	GGGTGAAATGGTAATTTTGGG	TGTGTTCAACGGGTGAGAAA
Chr1	SSR00927	138.913	20821600	CTgCTgAAAAggTgTTggCT	TggAAATgATCTAgCTgCCA
Chr1	SSR18335	155.618	20251684	CACCGATTTGAGCACCTACA	TGTCAATACAGGCAACACTCAA
Chr2	CM16	0	487540	TGCCTGTTGTGATTGAGGAG	TTCTTCTTACCTCCGCCAAA
Chr2	SSR05748	12.195	89863	TGTGGCCTGTGCTAAAATGA	TTTGGAAAAGCTAAAGCCCA
Chr2	SSR21090	23.865	1858848	CTGGAAATGGGGTTGGATTA	TGAATCAGCCGTTTCTGTGA
Chr2	SSR13504	52.278	4372184	CATCCCTTTTCCCTTCTTCTTT	TCCATGTGGAAGAATAACCCA
Chr2	SSR13532	64.737	5647445	AAACCCAAGAATTGTAAACCCA	TGATCCATTTCTATTCCTAACATTGA
Chr2	SSR23420	77.51	7873772	GAAGGGATGGTAGATGAAGGG	CTTCTCCCCTTTCTTGGCTT
Chr2	SSR04870	85.912	10118535	TACATGCCCCCTTGAAGAAG	TTCTTGGAGGATTTGCAGAAA
Chr2	SSR00030	89.002	10670350	TGAAATTGCTTACCCTTTGACC	CCATGTTTTGTAGGGATCGAG
Chr2	SSR21440	96.282	12743133	TTGTATCAAATTGTGCCTTAAAAGTT	TGCATGCTTATCTCCTTTTCA
Chr2	SSR04219	99.755	13713498	GAGACATTGTGGGCATTTGA	CTCATTTTCATCCAAAGGGC
Chr2	SSR01286	101.394	14658378	CCGAAAACCATTGTTCAAGC	TTTAGCTTAGTTTCCAAGCACTGA
Chr2	SSR13818	102.311	14974382	TTGTTAGTTCATTTGAGGTGTCAAG	TCCATATTAACTCTCTCAGGCTAACA
Chr2	SSR07278	111.088	17016846	ATCGCCATGGATTTCATCTC	CCACTCTTCGAGTACCGCTC
Chr2	SSR13131	130.428	18505180	AAAGCAGAGTATGGCATGGG	AAAAGCCAAAGAACCCAACA
Chr2	SSR16183	145.138	No Hits	GGAGAAATTTGATGGTGTAGCC	TTGCAAATCTCTAATACTTTGCCTT
Chr2	SSR15873	154.491	19269078	GCCAAAAATCCGAGAACAGA	TCCTGGGATTTTGATTTGCT
Chr2	SSR21276	170.2	21047286	TCGATGATAAATCCATCACATTTT	CGCTCTTGCCTATCTTGCTT
Chr2	SSR16462	185.348	22621989	TCACCTTCATGATTTTGCCA	GTCTTCAACGCAACCCAAGT
Chr3	SSR05312	0	2368898	GCCATGGAAAAGGTTTGAGT	TCAATTATGCCCCTTATCCC
Chr3	SSR21055	2.584	No Hits	CATCCTCATCCACCCAATCT	TCCAAACAAGTCTGACAAGGAA
Chr3	SSR17583	3.331	No Hits	GTTGATAAAAGAAGCCGCCA	TCTAAGCCGCCCTTAAAACA
Chr3	SSR00311	4.44	No Hits	TGAGGCAGAATCGAGAGTAGG	TGCAAAACTTCTTCTCGAATACA
Chr3	SSR20710	7.272	3610715	TAGAAGAAGCATCGGCAGGT	GAACTGGGGGTTTACCAGAA
Chr3	SSR16408	12.714	5630730	GTTGAATTATTGGAGGGATTTTT	CATTAATTTATTCAACCGTTTGGT
Chr3	SSR05891	25.315	No Hits	GTTTGGGTATAGGGAGACCG	TGAGATGTCGAGAACTCCATACA
Chr3	SSR04724	51.942	11217547	CTTTTCGCATTTCTTGACCA	CCACATTTCTAAGACCATGGTAGA
Chr3	SSR11633	77.851	15920930	AATGAGATAACATATGGATTTGTATGA	CACCGAACTGAAGTGTGCAG
Chr3	SSR02132	92.656	20792964	CAATTGGTATGAGTGAAAGATAAGC	CTCTGGTCCACCCAATCCT
Chr3	SSR12032	103.269	23296186	GGGGGTTTGAATTGAATGTG	CTCTTCTTTCCCCCTTTGCT
Chr3	SSR18428	119.278	26417833	CCATTCACTTCCTTTCCAGC	TGGTTTCAAGACCACCCTCT
Chr3	SSR21008	121.246	No Hits	TTGTCCACCTTGGATGTGAA	CCCACTTTTCCCTTTAACCC
Chr3	SSR01056	122.909	No Hits	AAAGGGAAAGGTAAATTGCCA	AGCAGTTCGGATGATATTGGA
Chr3	SSR17264	123.523	27811206	CCCTCCCACCTTCACTATCA	ATTGTCAACAAGAAACAGTGTGA
Chr3	SSR18141	126.932	28666375	ATGTCAATATGTGGGACCCG	TCGTAATAAATGGATGGCCC

Chr3	SSR02736	130.232	No Hits	ATGGGTGTGTTAGAAAGATTAGGA	CCAATTGATTTGGATTGGACA
Chr3	SSR05572	133.466	No Hits	GCAAACCATAAGTTTCCCCA	GATCGATATTGCAACGAATTACA
Chr3	SSR13949	136.079	No Hits	CCCATCAATCCCAGTTCATC	GTGCTGTTGCCATAAACCCT
Chr3	SSR16238	137.329	31163143	TCCGGATCTCTACCAAATGAA	TCAGAGCATAAAACCAGTGTGT
Chr3	SSR11594	139.091	31068516	GGCGAAAGATGAACAGAAGC	CCATGTTTTCGGTTTCCTTC
Chr3	SSR21304	142.913	No Hits	CCTCACCATGGTATTATCAATCG	GAATTCTTCTCGAATGTTTCCAA
Chr3	SSR21456	150.866	No Hits	AAGTGGGAAGGTTTGGAGGT	TTCCTTTTTCCCCCTTCATT
Chr3	SSR13974	152.46	32586332	ATGGAGTGTCAAGCAAGCAA	ATTGGGATTAGCAGCTTCCC
Chr3	SSR06011	153.532	No Hits	AGGGTCCAACTCAAACTCAAA	GCCCACCCTTAACATCTTACC
Chr3	SSR15124	161.296	No Hits	TCCACCCAATCTTTCCACTC	AGTGCTCTTCACATTTGCCA
Chr3	SSR13990	164.246	No Hits	CCAAATGCGGTGAAAAAGAT	GGGATTCTCTCTACCCCCAA
Chr3	SSR04628	165.078	No Hits	CCAACTTTTCGTAATGATTGAGAG	GTGAGCGTCAGAGCAATTCA
Chr3	SSR23177	165.672	No Hits	TGGATGAATGATGCCACAGT	CAAAAGCCTGTCTTGGTAAAAA
Chr3	SSR03066	173.006	37239584	CAAAACTTAAGGACCGAAAGGA	ACATGGTTGGTTAGTGGCCT
Chr3	SSR19521	173.316	No Hits	CAACCACACACAAATTCCCA	ATATGCCGCCCTAGAAAACA
Chr3	SSRw363	174.632	38975987	TGCCTTCTTCCTTTCCTTCA	CCAAACAACACCATCAGAAACA
Chr3	w2-8	175.1	No Hits	gAACCCAAATTggCATTAggT	gCATgTCTCTCTCCCTCAgC
Chr3	SSRW2-168	176.148	39694820	TGTCCTTCCCATTCAATCGT	CCACAGTTTGTGCATCCAAG
Chr3	SSR00733	178.079	No Hits	TCAAATTGTGATAGAGAGGCAGA	CCTCACAACCAAAAGGTCAAA
Chr4	SSR20307	0	No Hits	CCAAAAGGTCATTCGGTTCA	CGAAAAGTCAAGAAGAAACACAA
Chr4	SSR00012	4.97	No Hits	TCTCACCATGGTCACCTAATG	GGTCATTGAAGAGTCAAGTTGG
Chr4	SSR01949	6.938	823261	AGGAAAACCGGAAGCAGAAT	TCCACAGAACAACCGTGAAA
Chr4	SSR14026	7.379	No Hits	TACCGGAGAAATCATCGAGC	TCGCTAAACTCCAACACGAA
Chr4	SSR26406	7.541	5276352	AGCACACTTGGGTGGATTTC	CCTTTTCATTTGCAAACTTCC
Chr4	SSR23603	7.587	5973993	GACCATTTACCTTGCCATTTT	GAAAGCGAGCTAGGGCTTTT
Chr4	SSR19044	7.735	No hits	TgAAAAgAACATATggggCA	TgCAAACATAACAATTCgATCC
Chr4	SSR21730	7.887	No Hits	TTTTGTGCTGCTGATGAAGC	TTCTCATCGCAGGTGAAGTG
Chr4	SSR05005	10.095	10542520	CAGATTTAGCCAGTGGGTCC	ATCAGGGATCAGTCAATGGC
Chr4	SSR05783	12.182	3363484	AAAAGTCCCACAACATATGGAAA	TGGACAAACCAAGTCCCACT
Chr4	SSR19034	18.07	2972019	TCTTGCTTATCCCACCGTTC	GGGTTTCAAAACATTAACCCA
Chr4	SSR03481	28.122	12971131	TGTCTGTCCTTTTCCCTCCTT	AGAAACATGGTATGATATGTTGGA
Chr4	SSR07130	38.149	No Hits	CCACACACACACACAGTCACA	TCCCATTGTCCCTCACTCTC
Chr4	SSR17911	50.334	No hits	TGGAAGAGTGAGAAATGGGC	TGGGAGAAACGTTCTTTATTTTT
Chr4	SSR26165	51.919	No Hits	TCTTTTGTTGGTGAAAATTGAAA	CCTTCTCATGTGTATTGTCTTTTG
Chr4	SSR22862	52.641	No hits	AATTGGGTCAAAGAAAGGGG	TCGTCAAAATGAATCTCCGA
Chr4	SSR29080	54.698	No Hits	TTTTGCAATCAACCATTTTCC	GCCCATTCCCAATATATCCC
Chr4	SSR22155	55.8	22948132	AAACGGACATGTTAGGTGGC	TGGATTATTTAGGACAAACTGCAA
Chr4	SSR04454	56.572	16371689	GGAAAGTGTTGTGTTGCTCTTG	GATCCATTTGATGCATTGCT
Chr4	SSR21197	56.6	23134766	AAACCATTTGGTATTGGCGA	GTCCAACCAAACCAAGCCTA
Chr4	SSR18551	56.644	21268560	GATGTGCATGTGATCCAACAG	TGAATCTACTTGGGTTCGTTGTT
Chr4	SSR17406	56.645	No hits	GAGCCATCCATCAGAGAGAGA	ACCCACAAGCTTCAGAGGTC
Chr4	SSR01552	61.118	No hits	CACGACTTGCCATTAACCCT	CCCAAACTTTCCCCTCAAAT
Chr5	SSR19172	0	22085961	CGAGGAGAAGGACTACGACG	CGTGTGGAGCTTTCTCACAA
Chr5	SSR07184	13.851	No hits	CACCATTTTCCACAAAACGA	GCCATACCCTTCAACATAGGA
Chr5	SSR17464	15.767	21187949	ATCTCAGCCCTTGGATCCTT	TCTTCGAGAAATGGGATTTTG
Chr5	SSR14611	20.147	No hits	ATTTGGCTTCCAGGTTGATG	GAATCGAAAACCGATGAGGA
Chr5	SSR16691	22.454	9141668	CATGTTGAGTGGAAAAATTGAAA	TTCGTACATTGTCAAATGCG
Chr5	SSR23148	22.905	16426691	TGCCGTTTGGATCACATAGA	CAATACGCACAAAAAGCGAA
Chr5	SSR21280	23.231	7486163	CCATGATTTCAAATACCCCTT	TCTGTTTTCAAAATTGTTCGGA
Chr5	SSR18593	23.414	129876	TGGGCCTCAATCCTTGTCTA	ATAAGGTGTGTGGGCCAAAG
Chr5	SSR23132	23.558	15531345	GCCCTATCACTTCTTTGTTTTCA	CAAGAAATGGGGTGGAAGAA
Chr5	SSR16032	23.745	No hits	TCATGTCATCAATCACACCTTTC	TTTTTATCTTTTGTCGAGACTTGTTTT
Chr5	SSR11969	23.76	5973902	GGGTCATACCCAAAAGGGAG	TGCTTTAGCCGACAACTCAA
Chr5	SSR19836	23.778	No hits	TGCAGAAACATGAAAAGGGA	TGATTGAGTCCCATATGCCC
Chr5	SSR14269	23.87	13002103	GGAAATGGGAGATACACGGA	GCACTTTCCAGTTCCATGCT
Chr5	SSR01534	23.884	No hits	CTTCTGCTTCCGCTTCCTTA	GGAAGAAACCCACCAGAACA
Chr5	SSR15818	23.885	18966224	GGACATGTCAACTCCCCTGT	GCCTCTAGCCTGAAAGACCA
Chr5	SSR21291	23.9	16273295	CACTCCACAAGCAAACCAAA	CTATGGCGGAGAAGGAGTTG
Chr5	CSJCT315	23.913	No hits	CCACgAAATACAgATCAgCAAC	CACgTTACATTggACgAgAgAT
Chr5	SSR01610	23.945	No hits	TGGTTGTATATAGAAGGAAGATGTTGA	CCCTTTGAACGAAATCCAAA
Chr5	SSR19019	23.99	No hits	AgCTAgCTgCACCCACAACT	AAAgTATCCggAATCTTgTCAATC

Chr5	SSR14517	24.132	9375222	TCCCCATATGTTCTTCTCGC	AAGCTAAGGCAAACCAACCA
Chr5	SSR20895	24.414	No hits	CCTCTTCCTCCTCTTCCTCC	CCTTCCCCAATGTAGCAAAG
Chr5	SSR13340	24.532	No hits	GCTTTACATGGCTTCTCCCA	TTTTCCTTGGCGACGATTAG
Chr5	SSR06447	24.811	8580058	AAGTATGACGACACCCTTCG	CGCAAAACCGAAAGGTACATA
Chr5	SSR18729	25.669	13006047	TCCAATCCCACTCTGGTTTC	TCTTTCCAACACTAACCTTGGATT
Chr5	SSR15893	26.407	No hits	ATGGGAGCTACACGTTGAGG	ATAGGCTTCCATTCCAGGGT
Chr5	SSR07284	28.527	No hits	CTGCAGAATGTTTTGAGGCA	CCAACCTTGGAGGTGTTGAT
Chr5	SSR01498	38.314	22655145	GGCGCCACAAATATTCAACA	CCACAAACGTAAAGAGATTCACA
Chr5	SSR13620	57.781	24082650	TTTTCCGGTTCTACAATGGC	AGTTCAATCTTGTATGAGTGATATTGA
Chr5	SSRCSWCT	60.733	No hits	TTgAATTATgggTTCATTTTT	gACAATgATAAACTTCCCTgA
Chr5	SSR06303	67.05	24878262	AGCTCTCAACAACGAAGGGA	TGACTTTCTTGATGGTACCGC
Chr5	SSR03514	80.211	26718521	TAGGGTCCCCTTCCCTCATA	GGGTACCCAAAAGCAAGTGA
Chr5	SSR02459	89.88	27816197	TCGGAAGATGGGTTATTTGG	TGACCCCTCACATTCTCTCC
Chr6	SSR17962	0	776867	CAAAAGAAAAGCGCAAAAGG	GTCCCACTCCCAAAAACAGA
Chr6	SSR22275	2.058	1016060	ATACTTCGCCATTGACGCTT	TTACTAGGCGTTGTGGGAGC
Chr6	SSR02384	5.577	2783756	AAAAATCCGACAAATCGTGC	GGTCAAATGTTGCCTTTTGC
Chr6	SSR11343	9.365	4133077	GTGGGGTTGCTTTTGGATAA	CAATGGTTGCTTTGCTTCAA
Chr6	SSR01012	9.94	3855950	TCCAAAAATCGCGACCTAAA	GTGAGCCGTTGATTTCTCGT
Chr6	SSR02237	20.165	5124994	CTGAAAACTGGGGAATGGAA	GTTTGTTGCCTCGAAAAGGA
Chr6	CSJCT674	26.307	No hits	TAgAAAggAAgggATgTgATTAgg	ACAggTggTTAgAggTTAgAgCTg
Chr6	SSR24776	29.738	5812826	GTTCCTTGGTGCCATTGTTT	TCCAATTCCCTCTCTCTTCG
Chr6	SSR33284	38.229	6610818	TGGGAAATGGATAATTTGGTG	GGCATCCATCATTTCTTTGG
Chr6	SSR10954	49.454	9453905	TGCAAAACCAATTATTTGATATAGAGA	TTTCGGCAAAAGAACTAGGAG
Chr6	SSR21318	50.756	9372844	GACACCCCATTCCTCATCAT	GCTTCATCACTCCCAATTCAG
Chr6	SSRccap32	52.682	9764418	CGATCGTATTGATTAGATAAGCG	CGATTCGTCACTTTTCTTTCTTC
Chr6	SSR18405	53.077	10051003	CGCAGGTGCATCTCATGTAA	GACAAACAAGGGGACGAAAA
Chr6	SSRccap83	55.842	No hits	CgTgAACCTCCgATCTCATT	AgTCAAAgTTggCCAgTCgT
Chr6	SSR13884	58.198	11069339	GGATAATCCTGATTCCTGTGG	TGTGCAACTGAAAACGAAGC
Chr6	SSR16889	61.729	14857891	TCACATATTGGAATTTGAGGACA	AAGAGCAAACATTTGTTGAAACTC
Chr6	SSR18534	63.275	16606287	GCCCTGAAACTGAAGCTGAG	CTGATTGCCAAAGTCAAGCC
Chr6	SSR28064	65.136	16465180	GAAATTAATTAAGATCAAGAAAGGGA	ATCACTTGCATCACGTCTGG
Chr6	SSR18718	75.329	1611048	TGAAGCAAAGTAACCCCACA	CACAAATGGATTACAGAGCGAA
Chr6	SSR15955	76.709	18995075	TTTGAGCCTTGAGGCAAAGT	GCAATTCAACGTAATGGGCT
Chr6	SSR04252	83.129	20075416	AAAGAACACACATGGTGGTGG	AAGGAGTGTTTGAATAGGCCG
Chr6	SSR19165	89.004	21344610	AATCCACGTTGGTTGTCGTT	GAAGGGCCAAAAATGTTTCA
Chr6	SSR19970	101.362	23339816	TACCCGGTAATCTTCCCCTC	CAACAGAAGCAAAGTAGGTGGA
Chr6	SSR15482	107.356	24003956	AAAAACAGCAGGGCAGAAGA	GTTGAGCTGTCTATGGGGGA
Chr6	SSR15316	111.513	24577348	TCCAATTTTCTGGCGAAGAC	CAACCGAAACAACGTCTCCT
Chr6	SSR21115	118.335	25689547	GCAGCAGTCACCCAACCTAT	CACTGTGAACATATGGGAAGGA
Chr6	SSR02460	120.751	26252541	CTCAGAAACCCTTCCACCAA	CTGTACCGCGAGGACAGTTT
Chr6	SSR18251	136.478	28192598	AAATGGCAAAATAATGCATGG	GAGACAGCCACAGAGATTTGG
Chr6	SSR07477	138.205	29009111	CGTTTCATTCATTGCTGCAC	GCCCAGATTTGGTCATTTCTT
Chr6	SSR03357	138.624	28813523	AAAAGGGCAAGTCAAAACCC	GGGGAGGAAGAGAGACCCTT
Chr7	SSR30647	0	448536	GAAATGAGAGCAAGTTGCAAAA	AGGCGTAAATCTGACCGTTG
Chr7	SSR15432	13.611	4372675	TGAGAATTGGATGTGCGAAA	TTAGTTCATTGACATGGGATGAT
Chr7	SSR12994	14.856	2869178	TCATCCTGCAATGCAAAGAC	ACCGTGGATCTTAAGCCAGA
Chr7	SSR00084	15.226	40139	AAGTGTCAGCATGTCGTTCG	AAACGTCAGCCACACCTTTC
Chr7	SSR16591	33.436	12291486	TTTTGGGTAGAAATCAAGAAACG	GGGAATCAAACATTCATGGC
Chr7	SSR14861	40.786	12975963	ATTTCTTCCCCCACCAAAAC	ATGAATCCTCCTCCCAGAGC
Chr7	SSR22097	44.4	13483521	ACGGTCATCCGAATTCTCAG	CAACAAACGATCCAACATCG
Chr7	SSR06609	65.958	15169712	TCTAATGGAAACCCTCTCCG	GAGACGGAATCGGAATCAAA
Chr7	CSWCT11	71.228	No hits	ATAGGCAATATGGCTTCT	CACTTCAATGGAGTTTCG
Chr7	SSR23373	85.714	17467963	CCCACCATGCAAAACTCTTT	GTCTTTGCGCACCTCTCACT
Chr7	SSR23073	87.63	18153573	ATGAGGGAAGACTTTGGGGT	TTTCGTGAAGAAGGGGAAGA
Chr7	SSR00477	92.506	19197072	TATTGCGATGGTTTGACGTG	GCAAATTCCGGAGTTCGTTA
